# Shake-Down Spectroscopy as State- and Site-Specific Probe of Ultrafast Chemical Dynamics

**DOI:** 10.1021/jacs.5c09162

**Published:** 2025-09-02

**Authors:** Henry J. Thompson, Matteo Bonanomi, Jacob Pedersen, Oksana Plekan, Nitish Pal, Cesare Grazioli, Kevin C. Prince, Bruno N. C. Tenorio, Michele Devetta, Davide Faccialà, Caterina Vozzi, Paolo Piseri, Miltcho B. Danailov, Alexander Demidovich, Alexander D. Brynes, Alberto Simoncig, Marco Zangrando, Marcello Coreno, Raimund Feifel, Richard J. Squibb, David M. P. Holland, Felix Allum, Daniel Rolles, Piero Decleva, Michael S. Schuurman, Ruaridh Forbes, Sonia Coriani, Carlo Callegari, Russell S. Minns, Michele Di Fraia

**Affiliations:** † School of Chemistry and Chemical Engineering, 7423 University of Southampton, Southampton SO171BJ, U.K.; ‡ Dipartimento di Fisica, Politecnico di Milano, Milano 20133, Italy; § CNR–Istituto di Fotonica e Nanotecnologie (IFN), Milano 20133, Italy; ∥ Department of Chemistry, 5205 Technical University of Denmark, Kgs. Lyngby DK-2800, Denmark; ⊥ Department of Chemistry, Norwegian University of Science and Technology, Trondheim N-7491, Norway; # Elettra–Sincrotrone Trieste S.C.p.A., Basovizza 34149, Trieste, Italy; ¶ CNR–Istituto Officina dei Materiali (IOM), Basovizza, Trieste 34149, Italy; ∇ Faculty of Mathematics and Physics, Department of Surface and Plasma Science, Charles University, Prague 18000, Czech Republic; ○ Dipartimento di Fisica “Aldo Pontremoli”, Universitá degli Studi di Milano, Milano 20133, Italy; ⧫ CNR–Istituto di Struttura della Materia (ISM), Basovizza, Trieste 34149, Italy; †† Department of Physics, 3570 University of Gothenburg, Gothenburg 41296, Sweden; ‡‡ Daresbury Laboratory, Science and Technology Facilities Council (STFC), Warrington WA4 4AD, U.K.; §§ Linac Coherent Light Source, SLAC National Accelerator Laboratory, Menlo Park, California 94025, United States; ∥∥ J.R. Macdonald Laboratory, Department of Physics, 5308 Kansas State University, Manhattan, Kansas 66506, United States; ⊥⊥ Dipartimento di Science Chimiche e Farmaceutiche, Universitá degli Studi di Trieste, Trieste 34127, Italy; ## 6356 National Research Council Canada, Ottawa K1A0R6, Ontario, Canada; ¶¶ Department of Chemistry and Biomolecular Sciences, University of Ottawa, Ottawa K1N6N5, Ontario, Canada; ∇∇ Department of Chemistry, University of California, Davis, California 95616, United States; ○○ Center for Free-Electron Laser Science CFEL, Deutsches Elektronen-Synchrotron DESY, Notkestr. 85, 22607 Hamburg, Germany

## Abstract

Tracking the multifarious ultrafast electronic and structural changes occurring in a molecule during a photochemical transformation is a challenging endeavor that benefits from recent experimental and computational progress in time-resolved techniques. Measurements of valence electronic states, which provide a global picture of the bonding structure of the molecule, and core electronic states, which provide insight into the local environment, traditionally require different approaches and are often studied separately. Here, we demonstrate that X-ray pulses from a seeded free-electron laser (FEL) enable the measurement of high-resolution, time-resolved X-ray photoelectron spectra (XPS) that capture weak satellite states resulting from shake-down processes in a valence-excited molecule. This approach effectively combines the advantages of both valence- and core-state investigations. We applied this method to investigate photoexcited CS_2_ molecules, where the role of internal conversion (IC) and intersystem crossing (ISC) in determining the predissociation dynamics is controversial. We present XPS spectra from photoexcited CS_2_, obtained at the FERMI FEL. High-resolution measurements, compared to the corresponding spectra obtained from accurate multireference quantum chemical calculations, reveal that shake-down satellite channels are highly sensitive to both valence electronic and geometric changes. Previous studies of the predissociation dynamics have led to uncertain assignments of the branching between singlet and triplet excited states. We derive a propensity rule that demonstrates the spin-selectivity of the shake-downs. This selectivity allows us to unequivocally assign contributions from the bright and dark singlet excited states, with populations tracked along the predissociation dynamic pathway.

## Graphical abstract

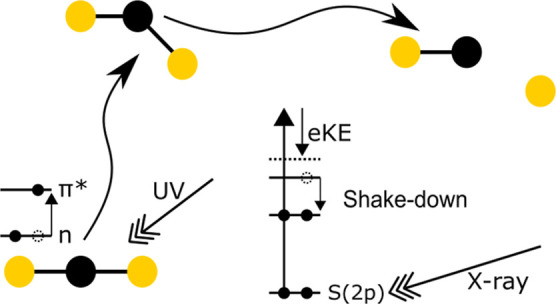

## 1 Introduction

Photochemical dynamics involve the coupled motion of electrons and nuclei that rearrange on ultrafast time scales. Even for a simple linear triatomic molecule, measuring and understanding the reaction mechanisms upon excitation is challenging. The ability to spectroscopically track photochemical processes occurring in a molecule on a femtosecond time scale is based on: the availability of suitable light sources, the sensitivity of experimental methods to the electronic and geometrical changes, and accurate theoretical protocols to interpret the results.

Among the various experimental probes available,^1^ photoelectron spectroscopies are particularly powerful and considered universal, since all states can in principle be ionized. Valence-shell photoelectron spectroscopy, where the binding energies of the outer (bonding) electrons are measured, is sensitive both to changes in the electronic character of the molecular states and to nuclear dynamics through changes in vibrational overlap.^2^ Core-shell photoelectron spectroscopy provides atomic site-specific information with the measured binding energy of the ejected electrons reporting on the chemical environment of the target element. Differences in binding energies reported in core-shell photoelectron spectroscopy provide chemical shifts that characterize different atomic environments and are a sensitive measure of local charge, with long established usage for solids^3^ and for ground-state molecules.^4^ More recently, this concept has been extended to electronically excited molecules,^5–13^ providing a sensitive probe of the change in local charge experienced by specific atoms within a molecule as it photochemically evolves.

X-ray photoelectron spectroscopy (XPS) measurements of excited-state molecules can also provide valence shell information through the observation of weaker secondary structures associated with satellite transitions such as shake-up or shake-down.[Fn fn1] In shake-up/-down, there is a rearrangement of the valence electrons as the core electron is removed. In the electronic ground state, molecules can undergo shake-up processes in which a valence electron is excited in conjunction with the core ionization transition. In such shake-up processes, the measured kinetic energy of the core-ionized electron is reduced, relative to that of the main photoline, by the energy associated with the valence excitation.

Upon core ionization of electronically excited states, the inverse process, shake-down, is possible.^14^ In shake-down, the populated valence excited state relaxes to a lower energy configuration, increasing the energy of the outgoing photoelectron by the corresponding amount (see sketch in panel 1­(b)). The observation of shake-down transitions from laser excited molecules and those undergoing dynamics remains largely unexplored. To observe such shake-down processes in a time-resolved experiment, highly stable and ultrafast X-ray pulses combined with highly sensitive electron detection are required. By performing high-resolution XPS measurements with the seeded FEL FERMI, we demonstrate the sensitivity of the XPS technique and the ability to measure shake-down satellite transitions through an investigation of the photodissociation of CS_2_. A schematic representation of the chemical dynamics and the photoionization processes is presented in Figure 1a,b respectively; throughout the manuscript we denote the electronic states with *C*_2v_[*D*_∞h_] labels.[Fn fn2] The measurements highlight the sensitivity and selectivity of the shake-down transitions to a particular spin multiplicity and allow us to spectrally isolate the singlet-state dynamics of the system.

**Figure 1:**
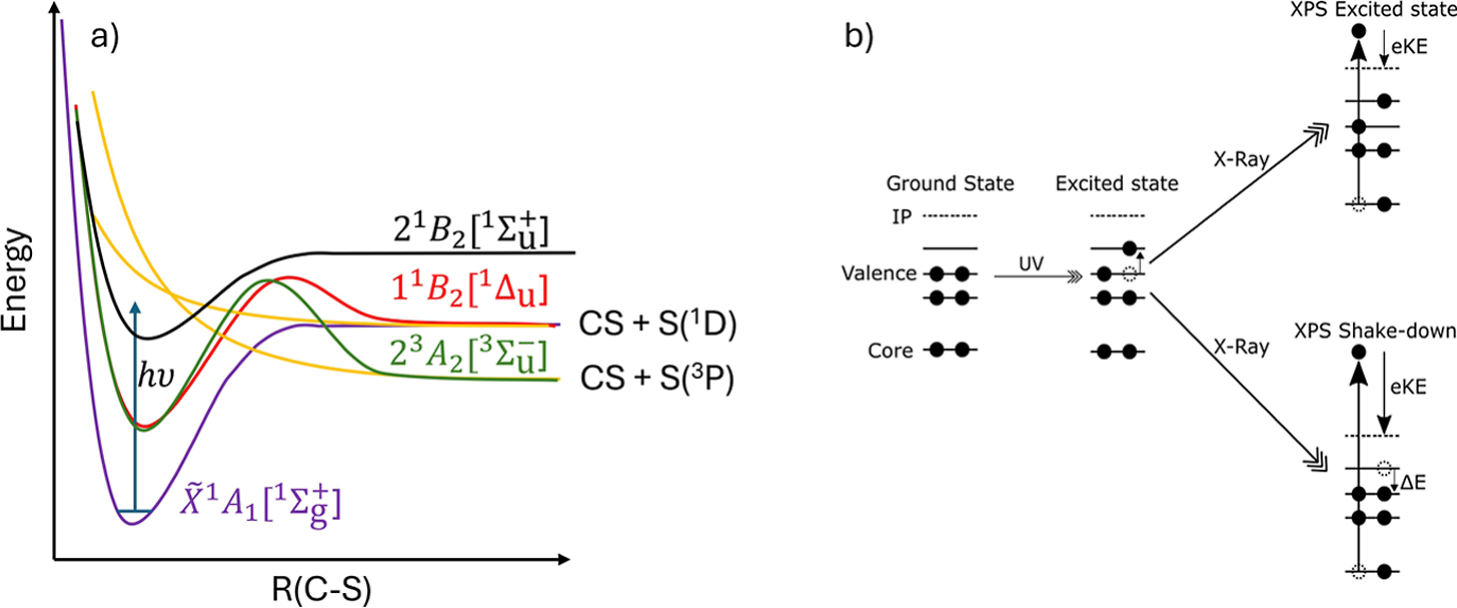
(a) Schematic representation of the potential energy surfaces of CS_2_ relevant to the photodissociation process. The sketch is based on the potentials presented in Smith et al.^11^ and Gabalski et al.^12^ Excitation with a UV pump photon populates the 2^1^ *B*_2_ [^1^Σ_u_^+^] excited state. Coupling to the manifold of singlet and triplet excited states leads to cleavage of one C–S bond and the formation of CS and S in either the ^1^D or ^3^P state. Energy level representations of the photoionization processes are shown in panel (b). A UV pump populates a valence excited state. Core ionization with an X-ray leads to peaks in the spectrum associated with ionization of the excited state, and to a secondary peak that has a higher electron kinetic energy (eKE) due to the relaxation of the valence excited state during the ionization process. As the molecular structure evolves on the excited state potential energy surface, the energy gap, Δ*E*, between the electronically excited and the ground state changes, leading to time-dependent shifts in the measured kinetic energy of the electrons associated with shake-down.

Previous measurements of CS_2_ have shown that, despite its structural simplicity, the dynamics of UV photolysis are complex, involving coupled nuclear and electronic motion, and two dissociation channels. Excitation around 6 eV (∼200 nm) leads to population of the 2^1^ *B*_2_ [^1^Σ_u_^+^] electronically excited state. Subsequent structural changes lead to the formation of a bent and stretched structure (as seen in scattering and Coulomb explosion measurements^15–18^ ) that facilitates internal conversion (IC) and intersystem crossing (ISC) processes, and the eventual formation of CS fragments in conjunction with a S atom. The S atom can be formed in either the ^1^D configuration in a spin-allowed process, or the ^3^P configuration in the spin-forbidden process. Repeated experiments have shown that, despite the subpicosecond ultrafast nature of the dissociation process, the spin-forbidden product dominates the yield. While this general picture is accepted, questions remain about which interactions control the branching between the singlet and triplet dissociation pathways. Conflicting assignment of existing photoelectron spectra also mean that it is unclear whether the triplet states of CS_2_ have any appreciable lifetime, or the dissociation is so rapid that the formation of products occurs before any population can be detected.^11,19,20^

Time-resolved XPS measurements of excited CS_2_ were recently reported.^12^ By probing the dynamics at the S 2p edge by means of SASE FEL pulses, Gabalski et al.^12^ were able to assign the main X-ray spectroscopic features to the dissociation products and follow the dynamics with reported time-scales in agreement with previous valence studies. In their paper, Gabalski et al. showcased the potential of the TR-XPS technique and made an initial attempt to track predissociation dynamics in excited CS_2_ molecules by means of core level ionization. Our use of a seeded FEL scheme, rather than a SASE FEL, offers a superior energy resolution and a negligible timing jitter, allowing one to distinguish weaker secondary photoemission processes and to capture the finer details of the dynamics. Utilizing the highly stable output of the two-stages seeded FEL at FERMI we obtain the valence and core spectrum in a single session, including the satellite states associated with shake-down processes. The satellite states, in particular, provide a sensitive and selective probe of the electronic and geometric structure changes that occur over the course of a photochemical reaction.

## 2 Results and Discussion

In Figure 2 we provide an overview of the experimental XPS data obtained following 6.2 eV (200 nm) excitation, and ionization with a 179.9 eV probe. The figure provides a map of the differential (pump-on minus pump-off) photoelectron intensity as a function of electron kinetic energy and time. The blue regions around 8.8 and 10 eV are due to depletion of the signals associated with the ionization of the S 2p_1/2_ and S 2p_3/2_ orbitals of the ground state of the CS_2_ molecule. Their kinetic energies correspond to binding energies of 171.08 and 169.81 eV, respectively.^21^ As the ground-state signal depletes, we see new, transient signals appearing at both higher and lower kinetic energies that decay on a sub-picosecond time scale. At longer delays, signals at even lower kinetic energy grow and reach a constant level within 2 ps of excitation, indicating that these relate to the CS and S dissociation products. The signals in the lower kinetic energy region show the same trends as those observed by Gabalski et al.^12^ but with greater energy resolution and signal-to-noise ratio. Signals at energies below 10.5 eV are therefore assigned as XPS photolines associated with the various reaction intermediates and product states created. At kinetic energies greater than 10.5 eV we see much weaker transient signals that cannot be assigned to direct X-ray ionization: these are related to shake-down transitions. In this higher kinetic energy range the signal is initially localized to energies ≲16 eV and its distribution shifts toward lower kinetic energies as a function of time.

**Figure 2:**
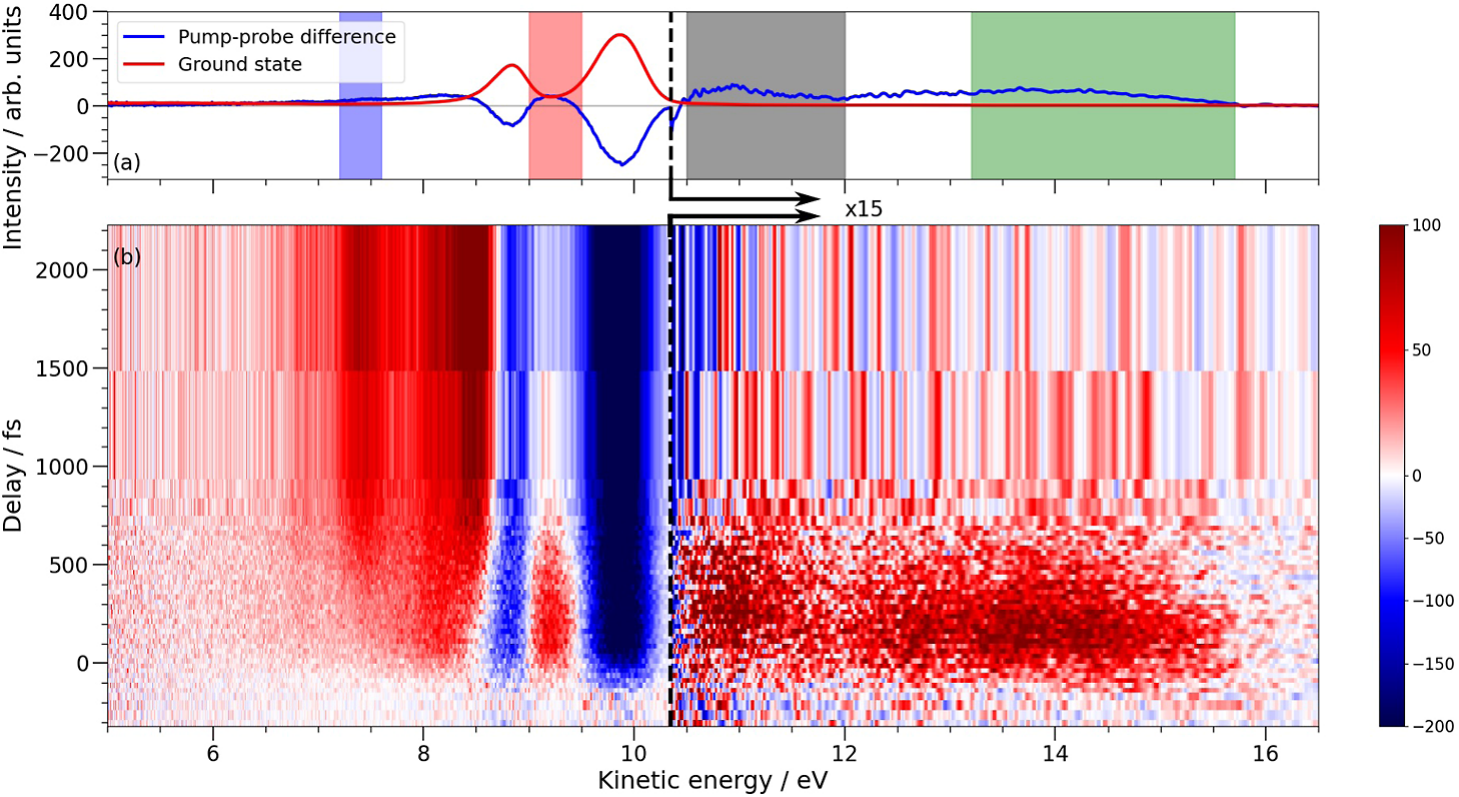
An overview of the time-resolved difference X-ray photoelectron spectrum of CS_2_ (b) obtained following 200 nm excitation, and ionization with a 179.9 eV probe with a comparison (a) of the ground state (red) spectrum scaled by 0.1 and the 0–600 fs pump–probe difference spectrum (blue). The color of the shaded regions corresponds with the integrated intensity of the same color within Figure 3.

To explore the transient nature of the signals appearing in various regions of the spectrum in a more quantitative manner, we plot the integrated intensity over a number of kinetic energy ranges in Figure 3. Panels (a) and (b) show the intensity profile of the shake-down transitions observed in the spectrum. We separately integrate over the 13.2–15.7 eV (a) and 10.5–12.0 eV (b) regions which show quite different temporal dynamics. The profiles show that the photoelectron intensity is initially localized in the higher kinetic energy range, (a). As this intensity decays, we observe a commensurate increase in intensity in (b), thereby indicating a flow of population between states. To extract lifetimes from the data, we fit the intensity profiles to a sequential kinetic model as schematically represented in Figure 3e (see Section S1.5 for more details and for the equations used), where excitation leads to population of the region (a), which subsequently decays into region (b). The fits are plotted as solid lines in Figure 3a,b and provide exponential time constants of 345 ± 12 fs and 167 ± 15 fs, respectively. These values match those previously obtained in valence photoelectron spectroscopy measurements of CS_2_ for the excited states populated en route to dissociation.^11,20^ At FERMI we have the advantage of being able to measure the XPS and valence photoelectron spectrum within the same session, and we obtain equivalent time constants in the time-resolved valence photoelectron spectrum (see Figures S6 and S7 in Section S2 for details).

**Figure 3:**
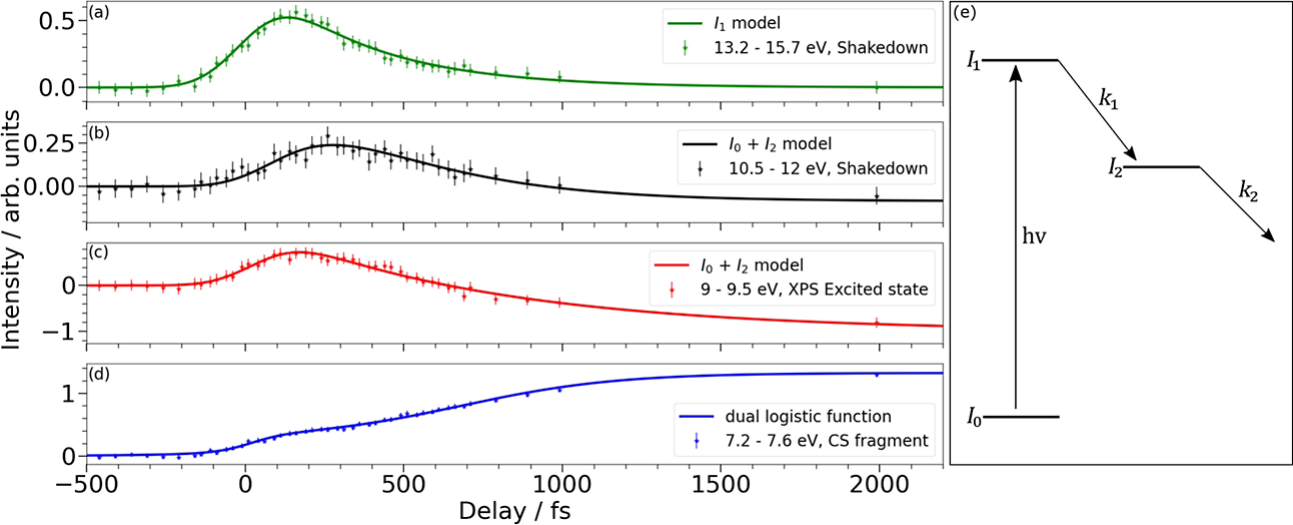
Integrated intensity of the photoelectron bands observed in the energy ranges 13.2–15.7 eV (a), 10.5–12.0 eV (b), 9.0–9.5 eV (c), and 7.2–7.6 eV (d). (e) Schematic representation of the kinetic model used to fit the experimental data. *I*_0_, *I*_1_ and *I*_2_ represent changes in state population associated with the laser excitation step, hν, and subsequent exponential decays of the populations in the two excited states defined by the rate constants, *k*_*n*_. The equations derived from the model and used to fit the data are given in Section S1.5.

Figure 3c shows the intensity profile for the transient increase in signal observed in the 9.0–9.5 eV range, that is, between the two spin-orbit split ground state depletion peaks. The transient enhancement observed in (c) is fit to an equation including ground state depletion and transient population of an excited state. The resultant fit shows that the decay of the transient excited state signal in panel (c) has the same temporal profile as the signals plotted in panels (a) and (b) combined. The common intensity profile indicates that the two energy regions, 9.0–9.5 and 10.5–15.7 eV, report on the same excited state populations but with the shake-down region showing greater spectral separation between components.

Figure 3d shows the signal associated with the formation of the CS dissociation product in the 7.2–7.6 eV range. The CS product signal in panel (d)-shows two time separated rises. We fit the data to a sum of two logistic functions to characterize these rises and obtain an appearance time, which defines the time at which 50% of the maximum intensity is obtained, as used in many time-resolved ion yield experiments. The logistic functions return appearance times of 20 ± 10 fs and 685 ± 23 fs with relative amplitudes of 0.25 and 1 for the fast and slow channels, respectively. The 685 fs appearance time matches the decay associated with the excited state signals observed in the shake-down and XPS regions of the spectrum.

To assign the peaks observed in the experiment, we computed the X-ray photoelectron spectrum of CS_2_ in the ground state and in all energetically accessible electronically excited states. The calculations are performed at the equilibrium geometry associated with that particular electronic state (reported in Section S3.1), with the results of all calculations and further details provided in the Supporting Information, see Sections S3.3 to S3.5. The theoretical XPS spectra of the initially populated valence excited state, as well as the identified IC- and ISC-accessible excited states, are plotted in Figure 4. Below the calculations are time-averaged experimental spectra obtained at early delays, where the various electronically excited states are populated, and at late delays where products have been formed. The calculated kinetic energies for all states are shifted by 0.76 eV to match the ground-state energies obtained in the experiment. No other scaling or corrections are applied.

**Figure 4:**
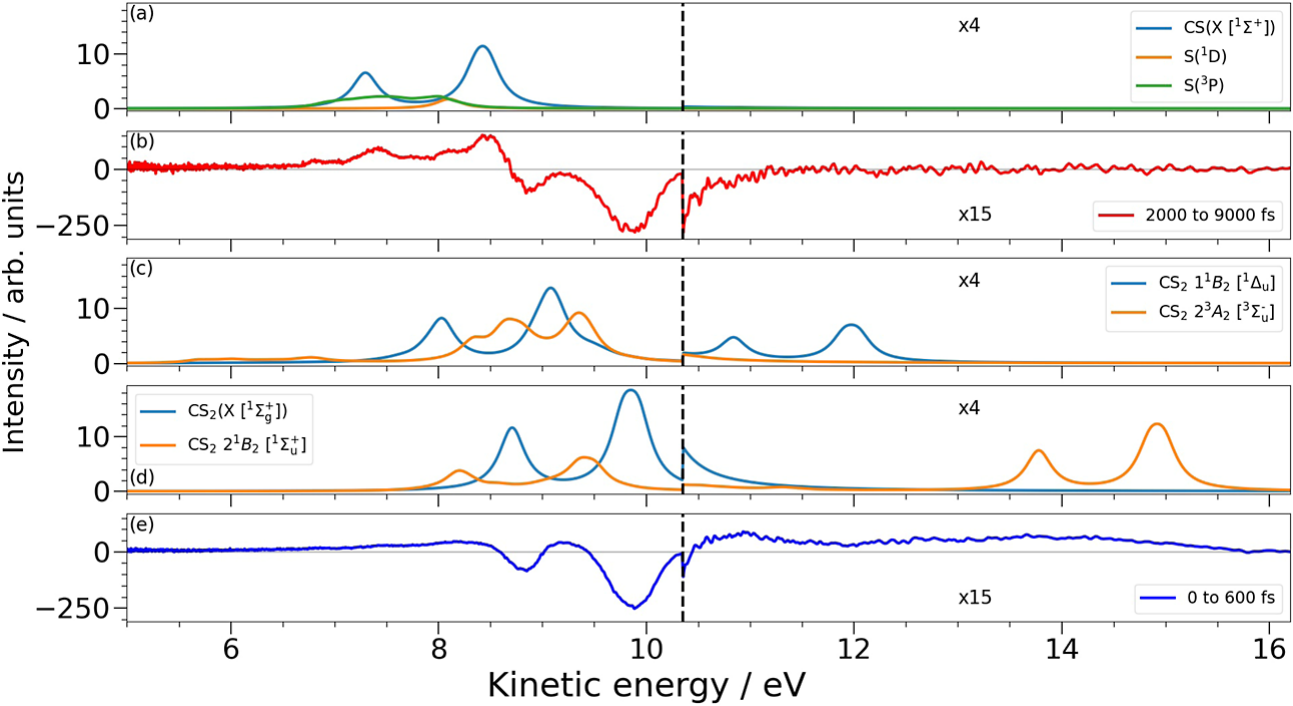
Comparison of theoretically calculated XPS spectra for the dissociation products (a), and the electronic states of CS_2_ (c) and (d) with the experimental spectra obtained at early (e) and late (b) delays. All calculated spectra have been shifted by 0.76 eV and broadened with Lorentzian functions with a full width at half-maximum of 0.3 eV.

Calculations of the spectra for the ground state, ^1^Σ_g_^+^, and the initially excited state, 2^1^ *B*_2_ [^1^Σ_u_^+^], Figure 4d, show that upon excitation the photoelectron kinetic energy associated with direct ionization of the electronically excited state shifts toward lower kinetic energy. This is explained by the π* ← *n* character of the transition that reduces the local electron density on the S atom, thereby increasing the binding energy of the S 2p electrons. The shift is relatively small, on the order of 0.5 eV, and leads to the appearance of the transient enhancement at kinetic energies between the two spin–orbit coupled peaks of the electronic ground state.

Ionization of the S 2p electron also gives rise to weaker signals at kinetic energies around 14–16 eV. These peaks are related to shake-down transitions where the valence hole is filled upon core ionization, leading to an increased kinetic energy of the outgoing electron. The peaks show an increase in kinetic energy relative to the core ionization peaks, with the energy gained by the electron being controlled by the energy gap between the valence excited state and the ground electronic state. The calculations of the ^1^ *B*_2_ [^1^Σ_u_^+^] electronic state are performed at the bent equilibrium geometry of the state, giving rise to the two sharp peaks associated with the two spin-orbit coupled states. We stress that in the experiment the molecule is known to asymmetrically stretch and bend such that a single dominant geometry is unlikely. As the energy spacing between the linear ground state, and the bent ^1^ *B*_2_ [^1^Σ_u_^+^] excited state, is heavily dependent on the structure, the energy gained upon shake-down is strongly geometry dependent. This leads to a smearing out of the photoelectron signal into the broad band in the 13–16 eV range observed in the experiment, which reflects the range of geometries probed at each delay.

Subsequent IC and ISC processes could lead to a number of energetically accessible states. To characterize which state or states may correlate with the shake-down signal seen in the 10.5–12.0 eV range, we calculate the expected XPS spectrum of the four lowest singlet and triplet excited states. The results of these calculations are plotted in Figures S18 and S19 in the Supporting Information and highlight that only the ^1^ *B*_2_ [^1^Δ_u_] state produces a signal in this region, and the calculated XPS spectrum of this state is shown in Figure 4c. The kinetic energy of the direct XPS signal has a very similar shape to that of the initially populated ^1^ *B*_2_ [^1^Σ_u_^+^] state, with a shift of only a few tenths of an eV expected due to the limited change in local charge density on the S atoms ionized. The shift in the shake-down region is, however, very large, on the order of 3 eV between the two states. This large shift can be understood in relation to the schematic potentials plotted in Figure 1a where the energy gap between the ^1^ *B*_2_ [^1^Σ_u_^+^] state and the ground state is much larger than that between the ^1^ *B*_2_ [^1^Δ_u_] state and ground state. The shifts predicted by theory match the experiment exceptionally well, suggesting that we can detect the different electronic states populated via the shake-down processes sensitively and selectively.

We also plot the expected spectrum for the ^3^ *A*_2_ [^3^Σ_u_^–^] state in Figure 4c as a characteristic spectrum for any triplet states that may be populated. Spectra for the other energetically accessible triplet states are provided in Figure S18 and have a very similar appearance that overlaps strongly with the singlet state XPS signals. This indicates that we cannot use the direct XPS signals to differentiate between the accessible electronically excited states. Critically, the triplet state spectra show no evidence of any shake-down transitions.

Because there is little exploration of shake-down processes for electronically excited molecules, some considerations are appropriate. In the sudden approximation, a monopole selection rule applies, such that the probability of a given transition is related to the squared overlap of the initial state with the ionized orbital removed and the final ionic state—that is, to the squared norm of the relative Dyson orbital. In the present case, the initial state is characterized by an excited φφ* electron pair, either singlet coupled (after the initial excitation) or triplet coupled (after ISC). The symbols φ and φ* refer, respectively, to occupied and virtual molecular orbitals in the closed-shell ground state. The shake-down state is characterized by an unpaired core electron in orbital φ_*c*_ (and a φφ̅ singlet electron pair). When computing the overlap, the role of the other “passive” electrons (i.e., the electrons not involved in the spin coupling of the final state) will give a similar contribution to all states, and the overlap will therefore be dominated by that of the three active electrons (the one unpaired core electron and the two electrons involved in the initial valence excitation). We show in Sect. 4.3, that only molecules that at the time of the core ionization event were in a singlet valence-excited state give rise to non-vanishing shake-down signals. In the primary ionization of the (singlet or triplet) valence excited state, the same electron pair (with the same spin coupling) appears in the final state, giving similar intensities for both couplings.

As CS_2_ has a singlet electronic ground state, we therefore expect to observe predominantly shake-down transitions originating from singlet excited states and not from triplet excited states. This general picture is seen in the shake-up spectrum of ground state, core-ionized CS_2_ of Wang et al.^22^ where the shake-up intensity of the triplet states is approximately an order of magnitude lower than that of the equivalent singlet state. This appears to be borne out by the theoretical predictions (see Section 4.3) and the experiment. While further study of shake-down is required, based on these arguments, we assign all of the signal in the shake-down region to singlet state populations.

Turning to longer delays and the formation of products, we present calculated spectra associated with the CS, and S (^1^D and ^3^P) fragments in Figure 4a, and in Figure 4b we show the experimental spectrum for late delays. The computed spectra agree extremely well with the experimental observation, reproducing the peak positions and relative intensities very well. We note that at early times there could be significant overlap between the signal associated with excited states of bound CS_2_ and that of the CS fragment peak appearing at kinetic energies around 8.5 eV. We therefore use the weaker of the two CS peaks at 7.5 eV to monitor the product appearance times, Figure 3d, as this appears to be free from any such contamination.

The combined experimental and theoretical results provide the following picture of the dynamics. Excitation into the ^1^ *B*_2_ [^1^Σ_u_^+^] state initiates bending and stretching of the nuclear framework. A minor amount of this population undergoes ballistic dissociation leading to formation of CS and S­(^1^D). The remaining population internally converts to the ^1^ *B*_2_ [^1^Δ_u_] state on a 350 fs time scale. The ^1^ *B*_2_ [^1^Δ_u_] state population decays on a 160 fs time scale, leading to CS + S fragments. The ability to directly assign specific excited states in the shake-down spectrum allows us to settle the controversy in the literature and assign the singlet intermediate state populated to the ^1^ *B*_2_ [^1^Δ_u_]. While spectral congestion means we cannot directly assign the dissociation product to either the singlet or triplet channel, previous studies show that the overriding majority of this decay should lead to the S­(^3^P) product. The measurements show that the decay of the excited singlet state signal observed in the shake-down region of the spectrum is on the same time scale as the formation of the CS products. One explanation could be that an unobserved triplet state intermediate is also populated and decays on an indistinguishable time scale. We find this suggestion unlikely given the dissociation limit for the triplet channel is approximately 1.1 eV lower than that for the singlet channel, and the shallower energy minimum of the triplet states compared to the singlet counterparts.^11^ We therefore conclude that any CS_2_ triplet state population is extremely short-lived, and that dissociation proceeds ballistically once intersystem crossing has occurred.

The proposed reaction mechanism can be summarized in the following scheme:
1a

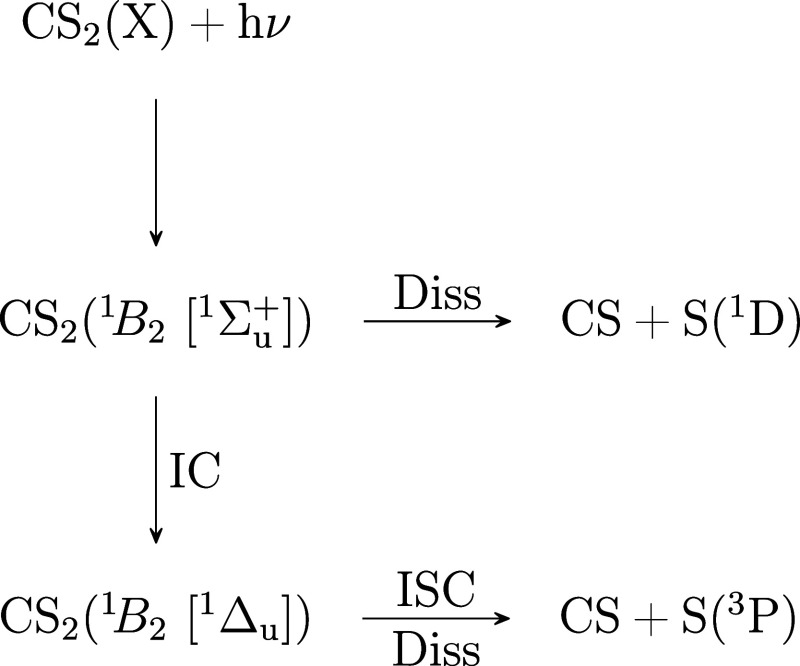




We highlight here that the proposed mechanism is based on the observation of the shake-down region of the excited state spectrum and of the product state XPS. Contrary to the direct XPS signals, where different electronic states produce highly overlapped peaks in the spectrum, analysis of the shake-down region allows us to exclusively monitor the singlet state population and distinguish the character of the populated excited electronic states. By monitoring the singlet state population and the product formation channels, we can draw a reasonable conclusion about the triplet states despite them not being observed directly. Based on the proposed mechanism, it appears likely that the instantaneous population of the triplet states of bound CS_2_ may be negligible such that probes that are highly sensitive to the triplet state population may struggle to detect these states.

## 3 Conclusion

We report the first time-resolved observation of shake-down transitions from laser-excited CS_2_ molecules probed above the S 2p edge with FEL radiation. In particular, upon excitation with 200 nm pump laser, we observed the shake-down signals associated with the 2^1^ *B*_2_ [^1^Σ_u_^+^] and the 1^1^ *B*_2_ [^1^Δ_u_] excited states. These peaks are significantly shifted from one another when compared to those observed in the direct XPS. The large shifts seen in the position of the shake-down peaks as the molecules change geometry and electronic state, allow us to unequivocally assign the states populated during the predissociation process and characterize the internal conversion from the bright excited state into the dark excited state. The delay in the appearance of the latter, with respect to the former, gives an indication of the time scale for the internal conversion to occur in excited CS_2_ molecules.

The results demonstrate the detailed and state-specific information that can be extracted from the shake-down process, over and above what could be extracted from the primary ionization alone. The large shifts in electron kinetic energy, related to the energy spacing between the valence excited state and the electronic ground state, provide a distinct observable that can be definitively assigned to specific states in the excited state manifold.

The observation of shake-down transitions opens a new spectroscopic window into the electronic structure changes that occur during a complex chemical reaction. The characteristics of the ground and excited states that lead to observable shake-down signals are currently unexplored, but lessons can be learned from static measurements of the shake-up process which will share the same selection and propensity rules. In principle, provided the transitions meet the symmetry selection rules, all states can be coupled. The cross-section of such transitions is, however, less clear and requires significant further exploration. The propensity of an allowed transition will significantly depend on the orbital overlap and the effective strength of the monopole/dipole with the states involved. We note here that the highest occupied molecular orbital that defines the start (end) point of the pump (shake-down) transition, in our particular study on CS_2_, is characterized as a lone pair orbital localized on the S atom. As such, this will have a strong interaction with the monopole derived from core ionization that is localized again on the S atom. Therefore, one might expect that ionization from a C core may lead to a lower propensity for shake-down. Such sensitivity, if demonstrated, provides a highly localized probe of the valence electronic state character which could enable tracking of the changing character of electronically excited states in much larger molecules.

## 4 Methods

### 4.1 Experimental Details

The experiment has been performed at the Low Density Matter (LDM) beamline^23^ of the Free Electron Laser FERMI^24^ in Trieste (Italy). The soft X-ray FEL probe pulse is generated in the FEL-2 machine,^25^ set to produce pulses at harmonic 12 of the seed laser wavelength, 248 nm, in the first stage, and harmonic 3 of the resulting 20.67 nm pulse (6.89 nm) in the second stage, at 50 Hz repetition rate; metal foil filters are available along the photon transport line to alter the balance of the two pulses. In particular, a palladium metal filter (Pd 100 nm) was used to abate the first-stage radiation. The FEL spot size was set to 50 μm (fwhm) and the pulse duration was estimated to be approximately 30 fs.

The pump laser setup is based on the availability of IR pulses, referred to as SLU (Seed Laser for Users) generated by a Ti:sapphire amplifier having the same laser oscillator as that used to drive the OPA generating the seed pulses.^26^ IR SLU pulses are optically transported to an optical table. The basic optical layout on this table is described in Ref. 27, and has subsequently been upgraded to include fourth harmonic generation (via sequential second-harmonic generation and sum-frequency generation). For this experiment, the UV excitation pulse was generated with a central wavelength of 199.72 nm and bandwidth of 0.86 nm fwhm (6.21 ± 0.03 eV); focus size 150 μm (fwhm); pulse duration 110 fs (fwhm); 25 Hz repetition rate. Up to 5 μJ of UV light could be generated, greatly exceeding the needs of the experiment (most measurements were done at 0.2 μJ, by reducing the intensity of the input IR). The SLU repetition rate was set to half of the FEL repetition rate to have FEL only shots and FEL + SLU shots to generate differential spectra in post-acquisition. The pump-probe instrument response function (IRF) was estimated by monitoring the XPS S 2p ground state depletion signals and resulted in an IRF of 108 ± 5.8 fs (for details, see the Supporting Information). When needed, the second stage of the FEL was “turned off” and the first stage alone at harmonic 5 of the seed wavelength at 261.1 nm was used to find and periodically check the temporal and spatial overlap of the FEL and SLU pulses. The resulting wavelength, 52.22 nm, was chosen because it coincides with the 1s5p ← 1s^2^ resonance of helium. The spatial and temporal optimization was done by monitoring the UV-induced ionization of helium atoms resonantly excited by the FEL pulse.

The basic layout of the endstation described in Ref. 28 does not include the Magnetic Bottle Electron Spectrometer (MBES), which became available as a later upgrade^29^ and was the spectrometer of choice in the present experiment, with the axis of the MBES oriented vertically. For the TR-XPS maps of Figure 2, a retardation voltage of 4 V was applied to increase the resolution in the region of the S 2p XPS peak. A mixture of 0.14 bar CS_2_ in 2 bar He was expanded into vacuum with a commercial pulsed valve (Parker Series 9, orifice aperture 250 μm) operated at the FEL repetition rate, 50 Hz, and nominal opening time 110 μs, in the endstation’s source chamber; a supersonic jet is formed along the horizontal long axis of the endstation, and passes through a conical skimmer (Beam Dynamics model 76.2, 3 mm diameter) into a differential pumping chamber, where it is further defined by a fixed-diameter iris (1.5 mm) and a set of piezoelectrically operated vertical slits (Piezosystem Jena PZS 3) before entering the detector chamber, where it crosses perpendicularly the FEL beam, the latter propagating along the horizontal short axis of the endstation. The SLU beam is sent into the detector chamber quasi-collinearly with the FEL (4° downward tilt). The absorption spectrum of CS_2_ measured by Hemley et al.^30^ suggests that the transitions excited by the SLU originate mainly from the vibrationally unexcited ground state, but with a small contribution from the level having one quantum of the bending mode. The upper levels populated will contain several quanta in the symmetric stretching and/or bending vibrational modes.

The calibration details of the spectrometer are outlined in the Supporting Information in particular Figures S2 and S5.

### 4.2 Computational Details

All initial and final states have been computed with the multistate restricted active space perturbation theory to second-order (MS-RASPT2) method, as implemented in OpenMolcas.^31^ *C*_2v_ point group symmetry was applied for the CS_2_ and CS molecules. Specifically, the linear CS_2_ and CS molecules were aligned along the *y* axis, while the bent CS_2_ molecules were oriented so that the *yz* plane constitutes the molecular plane, and the C_2_ rotation axis is oriented along the *z* axis. The *D*_2h_ point group symmetry was applied for the S atom. All calculations were performed with the ANO-RCC-VTZP basis set.^32^ Scalar relativistic effects were taken into account via the Douglas-Kroll-Hess Hamiltonian,^33^ and spin-orbit coupling via the a posteriori addition of the spin-orbit part of the DKH Hamiltonian as an effective mean-field one-electron operator.^34^

The orbital configuration of the ground state, including the lowest virtual orbitals, is reported in Table S7 in Supporting Information. The active space for CS_2_ was constructed with all the S 2p orbitals (symmetric 4a_1_, 5a_1_, and 1b_1_, and antisymmetric 3b_2_, 4b_2_, and 1a_2_) in the RAS1 subspace, followed by the occupied valence orbitals 7a_1_ (σ), 8a_1_ (π), 6b_2_ (σ), 7b_2_ (*n*), 2a_2_ (*n*), and 2b_1_ (π) and virtual orbitals 9a_1_ (π*), 10a_1_ (σ*), 8b_2_ (σ*), and 3b_1_ (π*) in the RAS2 subspace. The active space for CS was formed by placing the S 2p orbitals (4a_1_, 1b_1_, and 1b_2_) in the RAS1 subspace, and the occupied valence orbitals 5a_1_ (σ), 6a_1_ (*n*), 7a_1_ (σ), 2b_1_ (π), and 2b_2_ (π) as well as the virtual orbitals 8a_1_ (σ*), 3b_1_ (π*) and 3b_2_ (π*) in the RAS2 subspace. Lastly, the active space for S contained the 2p orbitals (1b_1u_, 1b_2u_, and 1b_3u_) in the RAS1 subspace, followed by the occupied 3s (3a_g_) and 3p (2b_1u_, 2b_2u_, and 2b_3u_) orbitals as well as the virtual 4s (4a_g_), 3p (3b_1u_, 3b_2u_, and 3b_3u_), and 3d (5a_g_, 6a_g_, 1b_1g_, 1b_2g_, and 1b_3g_) orbitals in the RAS2 subspace. The RAS3 subspace was kept empty, and we allowed for a maximum of one hole in the RAS1 subspace in all cases. The active orbitals are displayed in Section S3.2 of the Supporting Information (Figures S8–S18).

All XPS spectra were computed using the (state-specific) restricted active space state-interaction (RASSI) module,^35^ and we note that the Dyson amplitudes were calculated from biorthonormally transformed orbital sets,^36^ and using the spin-orbit coupled states. The final core-ionized states of the CS_2_ and CS molecules were obtained by state-averaging over 20 states per irreducible representation. For the S atom, we computed only one final core-ionized state per irreducible representation, except for the *A*_g_ irreducible representation, where we state-averaged over two final states. The core-ionized states were obtained by enforcing single electron occupation in the RAS1 subspace by means of the HEXS projection technique.^37^ An imaginary shift of 0.3 au was applied to all states in the second-order perturbation correction to avoid intruder states.

The XPS spectra of CS_2_ (Figures S18 and S19) were computed for the ground state (*X̃* ^1^ *A*_1_ [^1^Σ_g_^+^]) and for the following singlet valence-excited states 1^1^ *A*_2_ [^1^Σ_u_^–^], 2^1^ *A*_2_ [^1^Δ_u_], 1^1^ *B*_2_ [^1^Δ_u_], 2^1^ *B*_2_ [^1^Σ_u_^+^], and triplet valence-excited states 1^3^ *B*_2_ [^3^Σ_g_^+^], 1^3^ *A*_2_ [^3^Δ_u_], 2^3^ *B*_2_ [^3^Δ_u_], 2^3^ *A*_2_ [^3^Σ_u_^–^] where we use the notation *C*_2v_ [*D*_∞h_] ([*C*_*∞*h_] for CS). The XPS of CS_2_^+^ was computed for the ground state X̃ (^2^ *A*_2_ = ^2^ *B*_2_) [^2^Π_g_], whereas the XPS of CS was computed for the ground state *X̃* ^1^ *A*_1_ [^1^Σ_g_^+^]. Each XPS spectrum was constructed using initial and final states computed at the same (relaxed) structure. For each initial (ground or valence-excited) state XPS we used the optimized molecular structure of that specific state. The optimized structures are specified in the Supporting Information. The XPS of the S atom were computed for the ^1^D and ^3^P states. All resulting XPS spectra were averaged by the state degeneracy of the initial state. Lastly, the spin coupling between initial and final states has been taken into account by scaling the squared Dyson amplitudes of the singlet-to-doublet transitions by a factor of 2, those of doublet-to-triplet transitions by 3/2, and those of triplet-to-quartet transitions by 4/3.

### 4.3 Propensity Rule for the Shake-Down States

We propose a propensity rule stating that, as a general case, shake-down signals, such as those we observed here, will originate predominantly from molecules that at the time of the core ionization event were in a singlet state, whereas the participation of triplet valence-excited states is negligible.

In general, the appearance of satellite states can be discussed either with frozen or relaxed orbitals. Within the frozen orbital picture, the relaxation induced by the formation of a core hole may be described by means of single (and multiple, less intense) excitations with respect to the core-hole reference configuration. Alternatively, we may work directly with relaxed orbitals, which is the approach we adopt in the following.

The relative intensity of the photoionization event is typically approximated as the squared Dyson amplitude, see e.g. Ref. 38

$$I∼\sum_{\mathrm{c̃}=c,\mathrm{c̅}}|\left\langle \Psi_{final}\left|a_{\mathrm{c̃}}\right|\Psi_{initial}\right\rangle {|}^{2}$$ 1

where $a_{\mathrm{c̃}}$ is the annihilation operator that removes either an α or a β core electron. Ψ_initial_ and Ψ_final_ are the wave functions of the initial and final electronic states. In the following, standard barred notation will be used when referring to the β electrons. Hence, c̃∈{c,c̅} . The shake-down state amounts to a primary core ionization of the closed-shell ground state (e.g., the removal of a β core electron), and it may be written as $|\varphi_{c}\cdot \cdot \cdot \varphi \mathrm{\varphi ̅}\rangle$ .

The initial state may be a singlet or triplet valence-excited state, and may be written as

2 $$|\varphi_{c}{\mathrm{\varphi ̅}}_{c}\cdot \cdot \cdot {\left(\varphi \varphi^{*}\right)}^{S}\rangle \;\mathrm{or}\;|\varphi_{c}{\mathrm{\varphi ̅}}_{c}\cdot \cdot \cdot {\left(\varphi \varphi^{*}\right)}^{T}\rangle$$

where ${\left(\varphi \varphi^{*}\right)}^{S,T}$ denotes the spin symmetry of the electrons involved in the initial valence excitation (singlet or triplet). Specifically

3 $${\left(\varphi \varphi^{*}\right)}^{S}=\frac{1}{\sqrt{2}}\left(\varphi {\mathrm{\varphi ̅}}^{*}-\mathrm{\varphi ̅}\varphi^{*}\right)$$

4 $${\left(\varphi \varphi^{*}\right)}^{T}=\{\begin{array}{l} \varphi \varphi^{*}\\ \frac{1}{\sqrt{2}}\left(\varphi {\mathrm{\varphi ̅}}^{*}+\mathrm{\varphi ̅}\varphi^{*}\right)\\ \mathrm{\varphi ̅}{\mathrm{\varphi ̅}}^{*}. \end{array}$$

Hence, the relative intensities of the shake-down signals are found by computing

5 $$I_{S}=\sum_{\mathrm{c̃}=c,\mathrm{c̅}}\left|\langle \varphi_{c}\cdot \cdot \cdot \varphi \mathrm{\varphi ̅}|a_{\mathrm{c̃}}\right|\varphi_{c}{\mathrm{\varphi ̅}}_{c}\cdot \cdot \cdot {\left(\varphi \varphi^{*}\right)}^{S}\rangle {|}^{2}$$

6 $$I_{T}=\sum_{\mathrm{c̃}=c,\mathrm{c̅}}\left|\langle \varphi_{c}\cdot \cdot \cdot \varphi \mathrm{\varphi ̅}|a_{\mathrm{c̃}}\right|\varphi_{c}{\mathrm{\varphi ̅}}_{c}\cdot \cdot \cdot {\left(\varphi \varphi^{*}\right)}^{T}\rangle {|}^{2}$$

Note that the initial $|\varphi_{c}{\mathrm{\varphi ̅}}_{c}\cdot \cdot \cdot {\left(\varphi \varphi^{*}\right)}^{S,T}\rangle$ and final $|\varphi_{c}\cdot \cdot \cdot \varphi \mathrm{\varphi ̅}\rangle$ states are constructed from different orbital sets (hence non-orthogonal to each other), meaning that the overlaps above are computed as the determinant of the overlap matrix between the two orbital sets. Below we will omit explicit indication of the “passive” electrons (i.e., the electrons not involved in the spin coupling of the final state), and write the relative intensities as

7 $$I_{S,\mathrm{c̃}}=\left|\langle \varphi_{c}\varphi \mathrm{\varphi ̅}|a_{\mathrm{c̃}}\right|\varphi_{c}{\mathrm{\varphi ̅}}_{c}{\left(\varphi \varphi^{*}\right)}^{S}\rangle {|}^{2}$$

8 $$I_{T,\mathrm{c̃}}=\left|\langle \varphi_{c}\varphi \mathrm{\varphi ̅}|a_{\mathrm{c̃}}\right|\varphi_{c}{\mathrm{\varphi ̅}}_{c}{\left(\varphi \varphi^{*}\right)}^{T}\rangle {|}^{2}$$

The final shake-down state $|\varphi_{c}\cdot \cdot \cdot \varphi \mathrm{\varphi ̅}\rangle$ is a doublet state, and we assume in the following that $M_{S}=+\frac{1}{2}$ without loss of generality (the same reasoning applies to $M_{S}=-\frac{1}{2}$ ). Thus, only states (after the annihilation of one core electron) with the same spin projection can have a non-zero overlap with the shake-down state. It is not possible to obtain such a doublet spin projection by coupling a β core electron ${\mathrm{\varphi ̅}}_{c}$ with the singlet-coupled valence electrons ${\left(\varphi \varphi^{*}\right)}^{S}$ . Consequently

9 $$\begin{array}{l} I_{S,c}=\left|\langle \varphi_{c}\varphi \mathrm{\varphi ̅}|a_{c}\right|\varphi_{c}{\mathrm{\varphi ̅}}_{c}{\left(\varphi \varphi^{*}\right)}^{S}\rangle {|}^{2}\\ =|\langle \varphi_{c}\varphi \mathrm{\varphi ̅}|{\mathrm{\varphi ̅}}_{c}{\left(\varphi \varphi^{*}\right)}^{S}\rangle {|}^{2}=0 \end{array}$$

On the other hand, we may construct this doublet by removing a β core electron, in which case the relative intensity becomes

$$\begin{array}{l} I_{S,\mathrm{c̅}}={\left|\langle \varphi_{c}\varphi \mathrm{\varphi ̅}|a_{\mathrm{c̅}}|\varphi_{c}{\mathrm{\varphi ̅}}_{c}{\left(\varphi \varphi^{*}\right)}^{S}\rangle \right|}^{2}\\ =\frac{1}{2}{\left|\langle \varphi_{c}\varphi \mathrm{\varphi ̅}|\left(\varphi_{c}\varphi {\mathrm{\varphi ̅}}^{*}-\varphi_{c}\mathrm{\varphi ̅}\varphi^{*}\right)\rangle \right|}^{2}\\ \begin{array}{l} \approx \frac{1}{2}{\left|\left\langle \varphi_{c}|\varphi_{c}\right\rangle \left\langle \varphi |\varphi \right\rangle \langle \mathrm{\varphi ̅}|{\mathrm{\varphi ̅}}^{*}\rangle +\left\langle \varphi_{c}|\varphi_{c}\right\rangle \left\langle \varphi |\varphi^{*}\right\rangle \left\langle \mathrm{\varphi ̅}|\mathrm{\varphi ̅}\right\rangle \right|}^{2}\\ \approx \frac{1}{2}{\left|\langle \mathrm{\varphi ̅}|{\mathrm{\varphi ̅}}^{*}\rangle +\langle \varphi |\varphi^{*}\rangle \right|}^{2}={\left|\langle \varphi |\varphi^{*}\rangle \right|}^{2}\neq 0 \end{array} \end{array}$$ 10

where we approximated the state overlaps to leading order with respect to the orbital overlap matrix elements, and used the fact that ⟨φ_*c*_|φ_*c*_⟩ ∼ 1 and ⟨φ|φ⟩ ∼ 1.

In the case of an initial triplet valence-excited state, an α and a β core electron can only couple to the *M*_S_ = 0 and *M*_S_ = +1 spin projections of the triplet state to produce a doublet. Hence

$$\begin{array}{l} I_{T,c}={\left|\langle \varphi_{c}\varphi \mathrm{\varphi ̅}|a_{c}|\varphi_{c}{\mathrm{\varphi ̅}}_{c}{\left(\varphi \varphi^{*}\right)}^{T}\rangle \right|}^{2}\\ =|\langle \varphi_{c}\varphi \mathrm{\varphi ̅}|{\mathrm{\varphi ̅}}_{c}\varphi \varphi^{*}\rangle {|}^{2}\\ \begin{array}{l} \approx |\left\langle \varphi_{c}|\varphi^{*}\right\rangle \left\langle \varphi |\varphi \right\rangle \left\langle \mathrm{\varphi ̅}|{\mathrm{\varphi ̅}}_{c}\right\rangle {|}^{2}\\ \approx |\left\langle \varphi_{c}|\varphi^{*}\right\rangle \left\langle \mathrm{\varphi ̅}|{\mathrm{\varphi ̅}}_{c}\right\rangle {|}^{2}≃0 \end{array} \end{array}$$ 11

where the second equality follows from coupling a β core electron with the (*M*_S_ = +1)-component of the triplet state. Since the overlap between a core and valence orbital is expected to be very small, the product of such two overlaps will be vanishingly small. Lastly, we have

$$\begin{array}{l} I_{T,\mathrm{c̅}}=|\langle \varphi_{c}\varphi \mathrm{\varphi ̅}|a_{\mathrm{c̅}}|\varphi_{c}{\mathrm{\varphi ̅}}_{c}{\left(\varphi \varphi^{*}\right)}^{T}\rangle {|}^{2}\\ =\frac{1}{2}|\langle \varphi_{c}\varphi \mathrm{\varphi ̅}|\left(\varphi_{c}\varphi {\mathrm{\varphi ̅}}^{*}+\varphi_{c}\mathrm{\varphi ̅}\varphi^{*}\right)\rangle {|}^{2}\\ \begin{array}{l} \approx \frac{1}{2}|\left\langle \varphi_{c}|\varphi_{c}\right\rangle \left\langle \varphi |\varphi \right\rangle \left\langle \mathrm{\varphi ̅}|{\mathrm{\varphi ̅}}^{*}\right\rangle -\left\langle \varphi_{c}|\varphi_{c}\right\rangle \left\langle \varphi |\varphi^{*}\right\rangle \left\langle \mathrm{\varphi ̅}|\mathrm{\varphi ̅}\right\rangle {|}^{2}\\ \approx \frac{1}{2}|\left\langle \mathrm{\varphi ̅}|{\mathrm{\varphi ̅}}^{*}\right\rangle -\left\langle \varphi |\varphi^{*}\right\rangle {|}^{2}=0 \end{array} \end{array}$$ 12

where the second equality follows from coupling an α core electron with the (*M*_S_ = 0)-component of the triplet state, and the last equality is a consequence of the two contributions canceling out.

To summarize, eqs 9–12 show that only molecules in a singlet valence-excited state at the core ionization event will give rise to a non-vanishing intensity. We speak of a propensity rule rather than a strict selection rule because we only consider leading orders of the squared Dyson amplitudes. Moreover, we note that configuration interaction and spin-orbit coupling may complicate the picture and somewhat weaken our rule.

## Supplementary Material



## Footnotes

a Other processes such as shake-off are possible but we limit the discussion here for the sake of brevity.

b As pointed out in footnote 1 of Ref. 13, there is some inconsistency in the notation used in the existing literature for the state labels of CS_2_. Therefore, we will refrain from using the adiabatic state labels or the standard empirical (tilde) notation for the valence excited states.

## Notes

Time-resolved core experimental analysis details: spectrometer energy calibration, delay calibration, TR-XPS, bootstrapping analysis, fit equations & time constants. Time-resolved valence experimental analysis details: binding energy calibration, TR-UPS, kinetic fits. Computational information and results: geometries, active spaces, computed XPS spectra, assignment of spectroscopic features, additional CCSD results (PDF)
