# Supplementary material for: Shake-Down Spectroscopy as State- and Site-Specific Probe of Ultrafast Chemical Dynamics
Source: J Am Chem Soc. 2025 Sep 2;147(36):32851–60. doi: 10.1021/jacs.5c09162 (PMC12426938; doi:10.1021/jacs.5c09162)
Supplement: Supplementary file 1 [file ja5c09162_si_001.pdf]

Supporting Information:

Shake-down spectroscopy as state- and site-specific probe of ultrafast chemical dynamics

Henry J. Thompson,[†,∇∇∇] Matteo Bonanomi,[‡,¶,∇∇∇] Jacob Pedersen,[§,∥] Oksana Plekan,[⊥] Nitish Pal,[⊥] Cesare Grazioli,[#] Kevin C. Prince,[@,⊥] Bruno N. C. Tenorio,[§] Michele Devetta,[¶] Davide Faccialà,[¶] Caterina Vozzi,[¶] Paolo Piseri,[△] Miltcho B. Danailov,[⊥] Alexander Demidovich,[⊥] Alexander D. Brynes,[⊥] Alberto Simoncig,[⊥] Marco Zangrando,[⊥,#] Marcello Coreno,[∇] Raimund Feifel,[††] Richard J. Squibb,[††] David M. P. Holland,[‡‡] Felix Allum,[¶¶] Daniel Rolles,[§§] Piero Decleva,[∥∥] Michael S. Schuurman,[⊥⊥,##] Ruaridh Forbes,[¶¶,@@] Sonia Coriani,[*,§,△△] Carlo Callegari,[⊥] Russell S. Minns,[*,†] and Michele Di Fraia[*,#,⊥]

†*School of Chemistry and Chemical Engineering, University of Southampton, Southampton, SO171BJ, United Kingdom*

‡*Dipartimento di Fisica, Politecnico di Milano, 20133, Milano, Italy*

¶*CNR - Istituto di Fotonica e Nanotecnologie (IFN), 20133, Milano, Italy*

§*Department of Chemistry, Technical University of Denmark, Kgs. Lyngby, DK-2800, Denmark*

∥*Department of Chemistry, Norwegian University of Science and Technology, Trondheim, N-7491, Norway*

⊥*Elettra - Sincrotrone Trieste S.C.p.A., Basovizza, 34149, Trieste, Italy*

#*CNR - Istituto Officina dei Materiali (IOM), Basovizza, 34149, Trieste, Italy*

@*Faculty of Mathematics and Physics, Department of Surface and Plasma Science, Charles University, Prague, 18000, Czech Republic*

△*Dipartimento di Fisica "Aldo Pontremoli", Università degli Studi di Milano, 20133, Milano, Italy*

∇*CNR - Istituto di Struttura della Materia (ISM), Basovizza, 34149, Trieste, Italy*

††*Department of Physics, University of Gothenburg, Gothenburg, 41296, Sweden*

‡‡*Science and Technology Facilities Council (STFC), Daresbury Laboratory, Warrington, WA4 4AD, UK*

¶¶*Linac Coherent Light Source, SLAC National Accelerator Laboratory, Menlo Park, 94025, California, USA*

§§*J.R. Macdonald Laboratory, Department of Physics, Kansas State University, Manhattan, 66506, Kansas, USA*

∥∥*Dipartimento di Science Chimiche e Farmaceutiche, Università degli Studi di Trieste, Trieste, 34127, Italy*

⊥⊥*National Research Council Canada, Ottawa, K1A0R6, Ontario, Canada*

##*Department of Chemistry and Biomolecular Sciences, University of Ottawa, Ottawa, K1N6N5, Ontario, Canada*

@@*Department of Chemistry, University of California, Davis, 95616, California, USA*

△△*Center for Free-Electron Laser Science CFEL, Deutsches Elektronen-Synchrotron DESY, Notkestr. 85 22607 Hamburg, Germany*

∇∇∇*These authors contributed equally to this work.*

E-mail: soco@kemi.dtu.dk; r.s.minns@soton.ac.uk; difraia@iom.cnr.it

Contents

S1 Time-resolved core experimental and data analysis details S-3

S1.1 Spectrometer energy calibration S-3

S1.2 Delay calibration S-6

S1.3 TR-XPS S-7

S1.4 Bootstrapped analysis S-7

S1.5 Fit Equations & Time constants S-8

S2 Time-resolved valence experimental and data analysis details S-10

S2.1 Binding energy calibration S-10

S2.2 TR-UPS S-11

S2.3 Kinetic fits S-12

S3 Computational information and results S-14

S3.1 Geometries S-14

S3.2 Active Spaces S-16

S3.2.1 SCF Orbitals S-17

S3.2.2 RASSCF Orbitals S-20

S3.3 Computed XPS spectra S-23

S3.4 Assignment of spectroscopic features S-25

S3.5 Additional CCSD results S-26

S1 Time-resolved core experimental and data analysis details

Raw data were recorded on a shot-to-shot basis at the FEL repetition rate, 50 Hz, while the Seed Laser for Users (SLU) operated at a repetition rate of 25 Hz. As such, the structure of the data alternates sequentially between SLU-on and SLU-off. Raw time-of-flight (TOF) traces consist of the negative-going output signal of the magnetic bottle spectrometer, acquired by a CAEN VX1751 digitizer with 10-bit resolution, 1 V dynamic range, 1 ns sampling time. The sign of the digital traces is then inverted and a threshold of 3 mV above baseline is applied to each trace to reduce electrical noise. At every delay, the SLU-on and SLU-off spectra for each included shot are summed to produce separate delay-dependent SLU-on and SLU-off spectra. The SLU-on and SLU-off spectra obtained at each delay are subsequently normalised by the aggregate FEL pulse energy of the included shots. This process is followed for both the S 2p TR-XPS (Fig. S1) and the valence data sets. Note that the digitizer is triggered in advance of the FEL pulse, and the time of flight $\mathcal{T}$ is calculated as $\mathcal{T}_{\mathrm{raw}} - \mathcal{T}_0$, where $\mathcal{T}_0 \approx 5000$ ns is the arrival time of the FEL pulse; the spectra in Fig. S1 are plotted versus $\mathcal{T}_{\mathrm{raw}}$.

S1.1 Spectrometer energy calibration

The TOF axis is calibrated using the known peaks associated with ionisation of the ground state CS_2 molecules and the helium carrier gas. Measurements at an X-ray photon energy of 179.948 eV (6.89 nm) and spectrometer retardation voltages of 4 V and 100 V were used and a summary of the peaks used is given in Table S1. The kinetic energy values for the Auger-Meitner transitions were taken directly from[S1] and the retardation voltages were taken into account. Kinetic energy values for all other features were calculated using reported binding energies taking into account the photon energy and respective retardation voltage. These

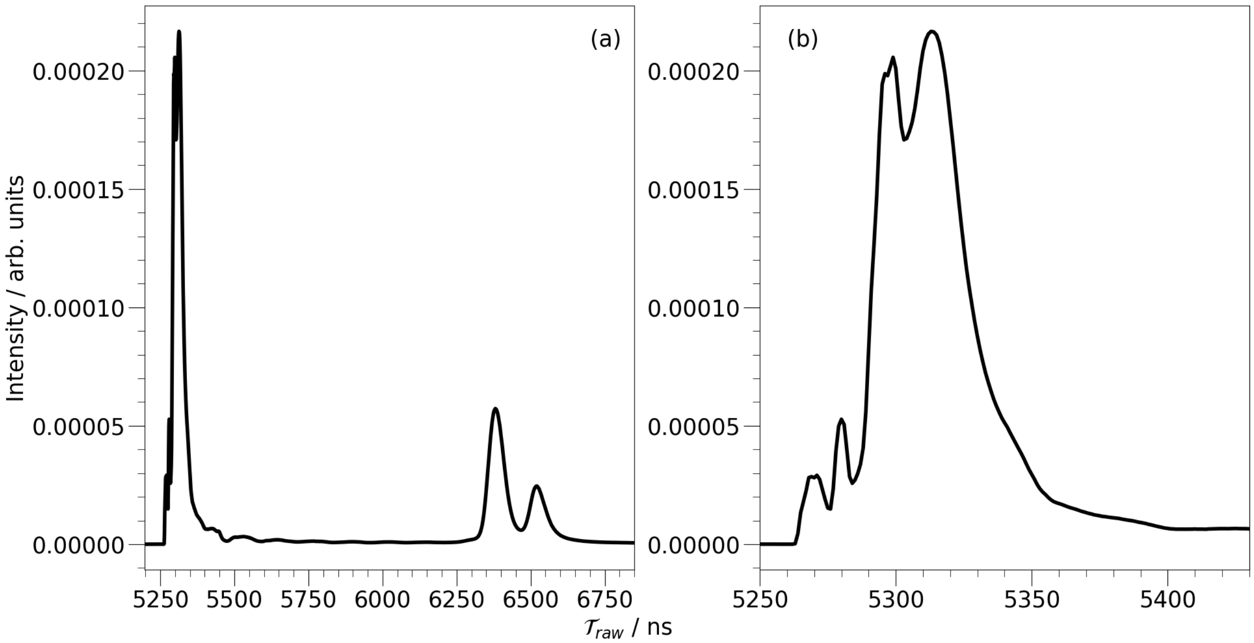

Figure S1: a) Time-of-flight photoelectron spectra of CS_2 probed using a 179.948 eV photon energy and a retardation voltage of 4 V. The S $2p_{3/2}$ and S $2p_{1/2}$ peaks that are the main focus of the work presented are located at TOF of 6380 and 6520 ns, respectively. The peaks between 5200 and 5360 ns relate to higher energy electrons associated with ionisation of the helium carrier gas, valence ionisation of CS_2 and secondary Auger-Meitner emission of CS_2 following core ionisation. An expanded view of the high energy region is plotted in b).

reference binding energy values for peaks originating from CS_2 were taken from,[S2] and,[S3] and for helium from.[S4]

The following equation is used to convert electron time-of-flight to kinetic energy:

$$
\begin{aligned}
E_k =& \frac{1}{2} m_\mathrm{e} \left(\frac{L}{\mathcal{T}}\right)^2 \\
=& \left(\frac{1686.065\ L/\mathrm{m}}{\mathcal{T}/\mathrm{ns}}\right)^2 \mathrm{eV}
\end{aligned} \tag{S1}
$$

where m_e is the mass of an electron, L is the flight length, and $\mathcal{T} = \mathcal{T}_\mathrm{raw} - \mathcal{T}_0$ is the electron time-of-flight, as defined above. Upon calibration of the equation to the data within Table S1, we obtain the best-fit values $L = 1.97 \pm 0.02$ m, $\mathcal{T}_0 = 5010 \pm 3.07$ ns.

The resulting energy calibration curve is presented in Fig. S2, plotted versus $\mathcal{T}_\mathrm{raw}$. Two curves are presented, giving the electron kinetic energy E_k as detected, taking into account the retardation applied, V_ret, and a second curve that defines the energy of the electrons $E_{\mathrm{k},0} = E_\mathrm{k} + |e|V_\mathrm{ret}$ at the point of ionisation ($e < 0$ is the value of the electron charge). The values presented in the manuscript are the kinetic energies of the electron upon ionisation, $E_{\mathrm{k},0}$, prior to the effect of the retardation voltage.

Following conversion to kinetic energy, the photoelectron-time-of-flight distribution is scaled by the Jacobian factor $|\mathrm{d}E_\mathrm{k}/\mathrm{d}\mathcal{T}| \propto E_\mathrm{k}^{-3/2}$ to obtain the correct photoelectron-energy distribution.

Table S1: Data used in the energy calibration of the magnetic bottle spectrometer.

Species	Feature	$\mathcal{T}_\mathrm{raw}$ / ns	E_k / eV	V_ret / V	Reference Features
CS_2	S $2p_{1/2}$	6520	4.848	4	$S(^2P_{1/2})$ [S2]
CS_2	S $2p_{3/2}$	6380	5.948	4	$S(^2P_{3/2})$ [S2]
CS_2	S $L_{2,3}$VV Auger peak	5521	42.1	100	$(\mathrm{S\ 2p})^{-1}\ ^2P_{3/2} \rightarrow (1\pi_g)^{-2}; a^1\Delta_g$ [S1]
He	1s $^2S_{1/2}$	5458	55.361	100	He 1s $^2S_{1/2}$ [S4]
CS_2	Valence onset	5406	69.868	100	$2\pi_g^{-1}$ (X $^2\Pi_g$) [S3]
CS_2	S $L_{2,3}$VV Auger peak	5295	138.1	4	$(\mathrm{S\ 2p})^{-1}\ ^2P_{3/2} \rightarrow (1\pi_g)^{-2}; a^1\Delta_g$ [S1]
He	1s $^2S_{1/2}$	5280	151.361	4	He 1s $^2S_{1/2}$ [S4]
CS_2	Valence onset	5268	165.868	4	$2\pi_g^{-1}$ (X $^2\Pi_g$) [S3]

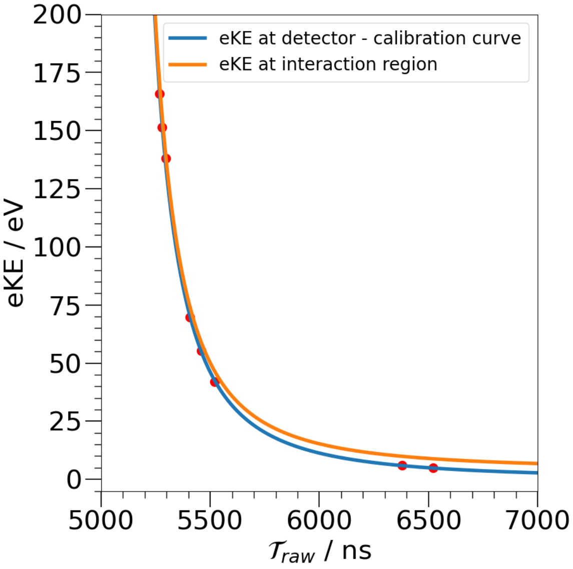

Figure S2: Time-of-flight to electron kinetic energy calibration curve resulting from the calibration points in Table S1. The red data points are the calibration points defined by the data in Table S1. The blue curve represents the fit of the measured kinetic energy at the detector position. The orange curve has the 4 V retardation voltage added to the energy and gives the kinetic energy of the electron upon ionisation, as displayed in the manuscript.

S1.2 Delay calibration

The time-zero for the pump-probe delay is experimentally defined by depletion of the ground state XPS signal located at an eKE between 9.5 eV and 10.2 eV. The integrated intensity over the $9.5-10.2$ eV range is plotted in Fig. S3 alongside a fit to an error function depletion. Due to overlapping contributions from excited state signals, the delays between 110 and 1900 fs are removed from the fit and are plotted in orange in Fig. S3. The fit to the remaining points provides a time-zero that is used in all plots and has an uncertainty of $\pm$ 4.1 fs. The fit also provides a pump-probe cross-correlation width (σ) of 108 $\pm$ 5.8 fs, consistent with the quadrature sum of the individual pulse durations. We use this value in the later kinetic fits.

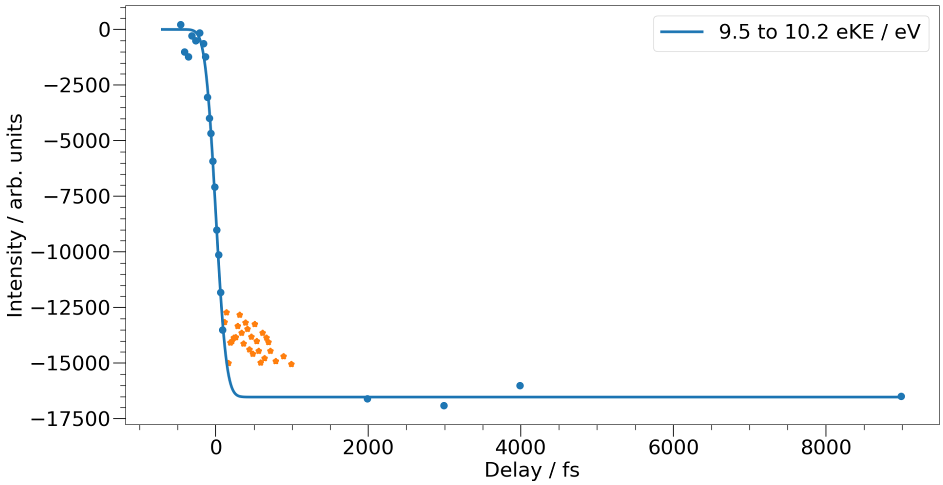

Figure S3: Time-dependent integrated intensity of the X-ray photoelectron spectrum of CS_2 between 9.5-10.2 eV. The blue and orange data points define the delay points that are included or excluded from the error function fit respectively.

S1.3 TR-XPS

In the manuscript we present the time-resolved data up to 2.3 ps. The full measurement covered a time range up to 9 ps. For completeness, in Fig. S4 we present the experimental TR-XPS data, over the full delay range measured, −0.46 to +9 ps. A Gaussian filter has been applied to the data using the `scipy.ndimage.gaussian_filter` function with a standard deviation of 1 (data point, 1 ns in TOF) for the Gaussian kernel applied along the energy axis. This process is applied to all experimental spectra and contour maps presented in this manuscript.

S1.4 Bootstrapped analysis

Confidence regions for the integrated intensities of the TR-XPS presented in Fig. 3 of the main manuscript were estimated via bootstrapping. By resampling the data on a shot-by-shot basis with replacement, allowing for repeats and omissions, a series of 5000 integrated intensities were obtained at each delay. From the distributions the standard deviations were obtained for each delay, with the 2σ range presented in Fig. 3 of the main paper.

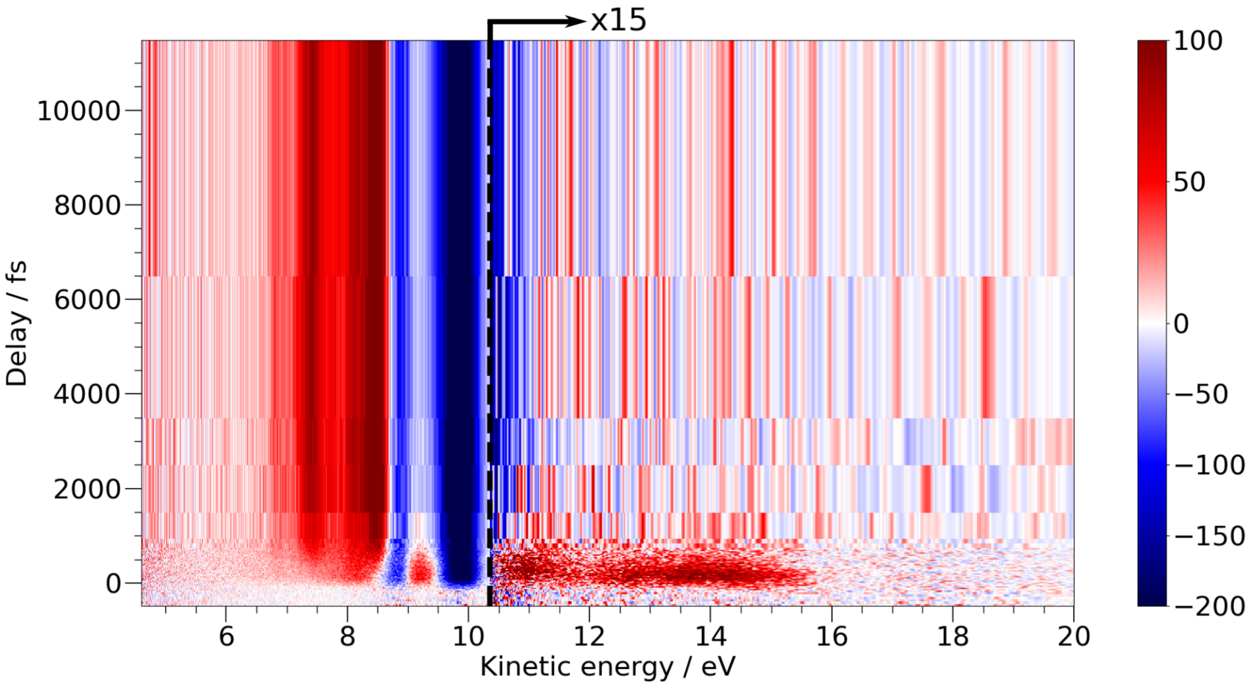

Figure S4: Time-resolved differential X-ray photoelectron spectrum of CS_2 obtained following 200 nm excitation and ionisation with a 179.9 eV probe.

S1.5 Fit Equations & Time constants

The integrated intensities plotted in Fig. 3 of the main paper are fit to various functions. Signals associated with changing populations of the ground and excited states of CS_2 are fit to a simple kinetic model that describes the flow of population between the ground and two separate electronically excited states. The principles of the model are outlined in panel (e) of Fig. 3 in the main manuscript. Interaction of the pump pulse with the molecule, transfers some population from the ground state, $|0\rangle$, to an electronically excited excited state, $|1\rangle$. State $|1\rangle$ exponentially decays with a rate constant, k_1, into a second electronically excited state, $|2\rangle$. Population in state $|2\rangle$ then exponentially decays with a rate constant of k_2. Assuming first order kinetics, equations that model the changes in state populations, I_0, I_1 and I_2, and therefore reflect changes in the signal intensities associated with these populations are given in equations S2 - S4 respectively.

$$I_0 = -A_0 \left(1 + \mathrm{erf} \frac{\Delta t}{\sqrt{2}\sigma}\right) \tag{S2}$$

$$I_1 = A_1 e^{-k_1 \Delta t} e^{\frac{(\sigma k_1)^2}{2}} \left[1 + \mathrm{erf}\left(\frac{\Delta t - \sigma^2 k_1}{\sqrt{2}\sigma}\right)\right] \tag{S3}$$

$$I_2 = A_2 \left\{ e^{-k_2 \Delta t} e^{\frac{(\sigma k_2)^2}{2}} \left[1 + \mathrm{erf}\left(\frac{\Delta t - \sigma^2 k_2}{\sqrt{2}\sigma}\right)\right] - e^{-k_1 \Delta t} e^{\frac{(\sigma k_1)^2}{2}} \left[1 + \mathrm{erf}\left(\frac{\Delta t - \sigma^2 k_1}{\sqrt{2}\sigma}\right)\right]\right\} \tag{S4}$$

where the A_n terms define relative amplitudes, Δt is the pump-probe delay, and σ defines the laser cross-correlation. σ has a fixed value of 108 fs in the fits as defined by the time-zero fit outlined in Section S1.2. The results of the fits are summarised in Table S2 and plotted in Fig. 3 of the main manuscript.

Table S2: Parameters obtained from fits to the measured shake-down and excited state XPS signals plotted in Fig. 3 (a) – (c) of the main paper.

Energy Range / eV	Fit Function	$(1/k_1)$ / fs	$(1/k_2)$ / fs
13.2 – 15.7	I_1	345.3 ± 11.6	N/a
10.5 – 12	$I_0 + I_2$	345.3 (Fixed)	166.8 ± 15.4
9 – 9.5	$I_0 + I_2$	20.8 ± 0.4	739.1 ± 34.2

The intensity of the CS product signal, I_{CS} is separately fit to a sum of logistic functions of the form given in equation S5.

$$I_{\mathrm{CS}} = \sum_n A_n \left[\frac{1}{1 + e^{-k_n\left(\Delta t - t_n^{\mathrm{offset}}\right)}}\right] \tag{S5}$$

where terms have the same meaning as above, k_n is the exponential time-constant, and t_n^{offset} is the offset delay from time zero. The sum is restricted to two terms in the XPS fit with the parameters extracted from the fit given in Table S3.

Table S3: Parameters obtained from the double logistic function fit to the CS fragment signal between 7.2˘7.6 eV. The fit is plotted along with the data in Fig. 3 (d) of the main paper.

Energy range / eV	t_1^{offset} / fs	$(1/k_1)$ / fs	t_2^{offset} / fs	$(1/k_2)$ / fs
$7.2 - 7.6$	19.7 ± 10.0	54.9 ± 16.7	685.5 ± 23.2	245.6 ± 19.5

S2 Time-resolved valence experimental and data analysis details

Time-resolved valence photoelectron spectroscopy (TR-UPS) data has also been recorded for comparison to previously published measurements of the valence photoelectron spectrum and to show consistency with the XPS measurements. As described in the experimental method section of the main manuscript, the FERMI FEL-2 radiation is produced in a double stage cascade configuration. To record valence data at higher resolution we "switch off" the second stage, obtaining radiation at 20.67 nm (59.98 eV) with the first stage alone. Importantly the pump conditions were identical to those used in the XPS measurements reported in the main manuscript.

S2.1 Binding energy calibration

The valence photoelectron spectroscopy measurements are plotted against binding energy to aid comparison with the existing literature. The calibration of the spectrometer followed the same procedure as outlined for the XPS measurements, but used the known valence ionisation peaks outlined in Table S4, with literature values taken from[S3] and .[S4] A retardation voltage (35 V, nominal) was applied to improve spectral resolution. Based on Einstein's photoelectric

Table S4: Time-of-flight to binding energy calibration points used in the calibration of Fig. S5.

Feature	$\mathcal{T}_{\mathrm{raw}}$ / ns	V_{ret} / V	Reference features
X	5883	35	$2\pi_{\mathrm{g}}^{-1}$ (X $^2\Pi_{\mathrm{g}}$) [S3]
A	5980	35	$2\pi_{\mathrm{u}}^{-1}$ (A $^2\Pi_{\mathrm{u}}$) [S3]
B	6037	35	$5\sigma_{\mathrm{u}}^{-1}$ (B $^2\Sigma_{\mathrm{u}}^{+}$) [S3]
C	6127	35	$6\sigma_{\mathrm{g}}^{-1}$ (C $^2\Sigma_{\mathrm{g}}^{+}$) [S3]
Helium	8640	35	He 1s $^2\mathrm{S}_{1/2}$ [S4]

equation, and on Eq. S1, we define the photoelectron binding energy as:

$$E_{\mathrm{b}} = hv - \frac{1}{2}m_{\mathrm{e}}\left(\frac{L}{\mathcal{T}}\right)^2 - |e|V_{\mathrm{ret}} \tag{S6}$$

Upon fitting equation S6 to the data in table S4, we obtain best fit values to be: $L = 1.99 \pm 0.14$ m, $\mathcal{T}_0 = 5030 \pm 60.54$ ns, $V_{\mathrm{ret}} = 34.52 \pm 0.29$ eV, with the FEL photon energy $h\nu$ fixed at the nominal value, 59.98 eV. The resulting TOF to binding energy conversion is plotted in Fig. S5.

S2.2 TR-UPS

In Fig. S6 we present the differential, pump-on minus pump-off, valence photoelectron spectra obtained at pump-probe delays out to 9 ps. Blue regions in the spectra relate to depletions of the ground state signal, while the red regions are enhancements due to excited state populations (between 3.2-9.5 eV) or product state formation (between 11.4-14.1 eV). The time-zero point for the pump-probe delay is defined by the kinetic fit (eq S3) to the signal associated with population of the initial excited state as measured at electron binding energies between 3.2 – 7.5 eV.

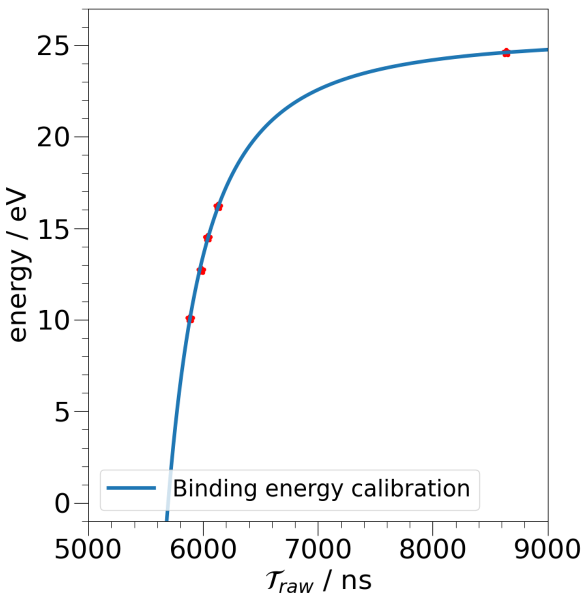

Figure S5: Time-of-flight to binding energy calibration curve (blue line) resulting from the calibration points in Table S4 (red dots).

S2.3 Kinetic fits

As with the analysis of the XPS data we extract time-dependent intensity profiles for various energy regions to explore the time-scales and kinetics of the changes observed.

In line with previous work,[S5,S6] we take the signals observed at binding energies below 9.5 eV as indicative of electronically excited states. Like the shake-down signals seen in the XPS measurements, the excited state signal in the valence measurement is split into two spectrally and temporally separate regions. The intensity profiles associated with the 3.2 – 7.5 eV and 8.0 - 9.5 eV binding energy regions are plotted in panels (a) and (b) of Fig. S7 respectively. To directly compare the time-dependence of the valence signals with those in the shake down region, we fit the valence data to the same kinetic equations outlined in Section S1.5. The lower binding energy region, 3.2 – 7.5 eV, is fit to equation I_1, representing the initial excited state population, Fig. S7(a), while the higher binding energy region, 8.0 - 9.5 eV, is fit to equation I_2 as a secondary populated region, Fig. S7(b). The extracted fit parameters are reported in Table S5. The extracted time constants, $1/k_1$ and $1/k_2$, were

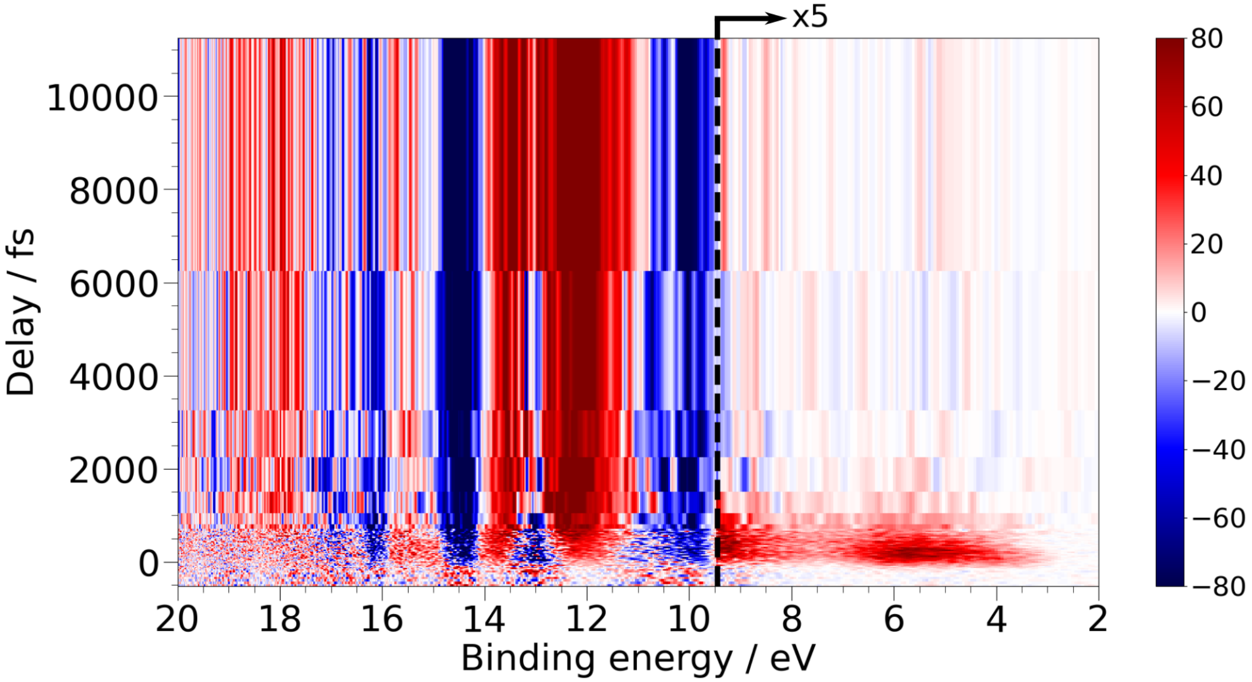

Figure S6: Time-resolved differential UV photoelectron spectrum of CS_2 obtained following 200 nm excitation and ionisation with a 60 eV probe up to 9 ps.

422.2 ± 26.5 fs and 143.6 ± 14.1 fs respectively, which closely match the values obtained from the shake-down regions indicating they derive from the same excited state populations.

Table S5: Parameters obtained from fits to the measured excited state UPS signals plotted in Fig. S7

E_b range / eV	σ / fs	$(1/k_1)$ / fs	$(1/k_2)$ / fs
3.2 – 7.5	109.6 ± 6.2	422.2 ± 26.5	N/a
8.0 – 9.5	109.6, fixed	422.2, fixed	143.6 ± 14.1

Time constants for all of the valence fits are presented in Table S5. These values match those previously obtained in valence photoelectron spectroscopy measurements of CS_2,[S5,S6] confirming that the general experimental approach is consistent with previous works.

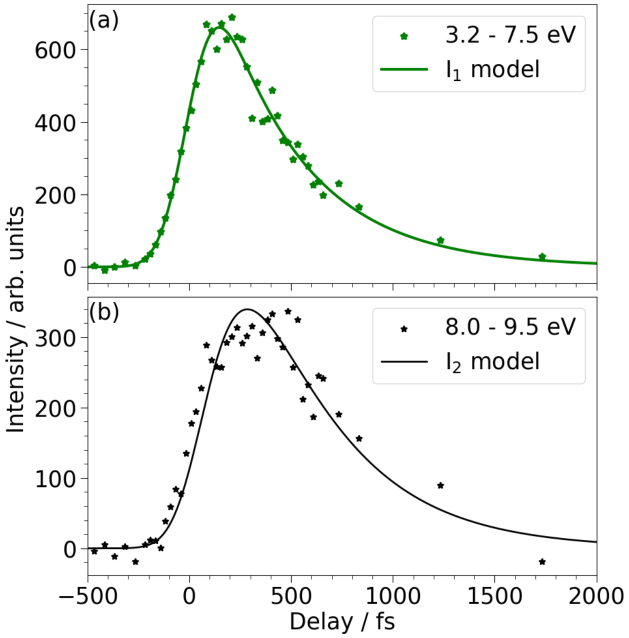

Figure S7: Integrated photoelectron intensity of the Valence photoelectron bands observed at 3.2 – 7.5 eV (a) and 8.0 – 9.5 eV (b) (Binding energy). Each intensity profile is fit to the equations described in the above SI text.

S3 Computational information and results

S3.1 Geometries

The ground-state equilibrium structure of CS_2 was taken from Ref. S7. The excited-state geometries of CS_2 have been optimized at the (frozen-core) EOM-CCSD/aug-cc-pVDZ level of theory. The structure of CS_2^+ was optimized using (frozen-core) EOM-IP-CCSD/aug-cc-pVDZ. All geometry optimizations have been performed with Q-Chem (version 6.1)[S8] with default convergence criteria and enforcing $C_{2\mathrm{v}}$ point group symmetry. The ground-state structure of CS was taken from Ref. S9. The bond lengths and bond angles of CS_2, CS_2^+, and CS are tabulated in Tab. S6.

Table S6: Bond lengths and bond angles of CS_2, CS_2^+, and CS.

Molecule	State	R_{CS} [Å]	θ_{SCS} [deg.]	Taken from
CS_2	GS	1.555000	180.000000	Ref. S7
	$1\ ^1A_2$	1.651757	137.092077	This work
	$2\ ^1A_2$	1.633878	176.234559	This work
	$1\ ^1B_2$	1.653275	129.426236	This work
	$2\ ^1B_2$	1.671133	150.501754	This work
	$1\ ^3A_2$	1.653716	136.278039	This work
	$2\ ^3A_2$	1.632599	166.253898	This work
	$1\ ^3B_2$	1.650480	124.773319	This work
	$2\ ^3B_2$	1.627617	168.543120	This work
CS_2^+	GS	1.575570	180.000000	This work
CS	GS	1.534942	180.000000	Ref. S9

S3.2 Active Spaces

The Hartree-Fock electronic configuration of CS_2 at linear and bent geometry is summarized in Table S7.

Table S7: Electronic configuration (Hartree-Fock) of the GS in linear and bent geometry.

$D_{\infty h}$	D_{2h}	C_{2v}	Assignment
$(1\sigma_u)^2$	$(1b_{1u})^2$	$(1b_2)^2$	S 1s
$(1\sigma_g)^2$	$(1a_g)^2$	$(1a_1)^2$	S 1s
$(2\sigma_g)^2$	$(2a_g)^2$	$(2a_1)^2$	C 1s
$(2\sigma_u)^2$	$(2b_{1u})^2$	$(2b_2)^2$	S 2s
$(3\sigma_g)^2$	$(3a_g)^2$	$(3a_1)^2$	S 2s
$(3\sigma_u)^2$	$(3b_{1u})^2$	$(3b_2)^2$	S 2p
$(4\sigma_g)^2$	$(4a_g)^2$	$(4a_1)^2$	S 2p
$(1\pi_g)^4$	$(1b_{2g})^2$, $(1b_{3g})^2$	$(4b_2)^2$, $(1a_2)^2$	S 2p
$(1\pi_u)^4$	$(1b_{2u})^2$, $(1b_{3u})^2$	$(1b_1)^2$, $(5a_1)^2$	S 2p
$(5\sigma_g)^2$	$(5a_g)^2$	$(6a_1)^2$	σ
$(4\sigma_u)^2$	$(4b_{1u})^2$	$(5b_2)^2$	σ
$(6\sigma_g)^2$	$(6a_g)^2$	$(7a_1)^2$	σ
$(5\sigma_u)^2$	$(5b_{1u})^2$	$(6b_2)^2$	σ
$(2\pi_u)^4$	$(2b_{2u})^2$, $(2b_{3u})^2$	$(2b_1)^2$, $(8a_1)^2$	π
$(2\pi_g)^4$	$(2b_{2g})^2$, $(2b_{3g})^2$	$(7b_2)^2$, $(2a_2)^2$	n
$3\pi_u$	$5b_{2u}$, $3b_{3u}$	$3b_1$, $9a_1$	π^*
$7\sigma_g$	$7a_g$	$10a_1$	σ^*
$6\sigma_u$	$6b_{1u}$	$8b_2$	σ^*

We show in Figs. S8 to S16 the active spaces used in our calculations for the ground state and the valence excited states of CS_2. The active orbitals of CS are shown in Figs. S12 and S17. The orbitals are plotted with Jmol[S10] using an MO cutoff and translucent thresholds of 0.02 and 0.25, respectively. We provide both the SCF and RASSCF orbitals since the appearance of the $10a_1$ and $8b_2$ orbitals changes significantly between the two sets of orbitals. We have kept the SCF orbital labels for the RASSCF orbitals. The basis set used is ANO-RCC-VTZP basis set. The RAS1 space consists of the six S 2p occupied orbitals (12 electrons). The RAS2 space consisted of 10 orbitals (6 occupied and 4 virtual at SCF level) and 12 electrons. RAS3 is empty. We allowed for a maximum of one hole in the RAS1

subspace in all cases.

S3.2.1 SCF Orbitals

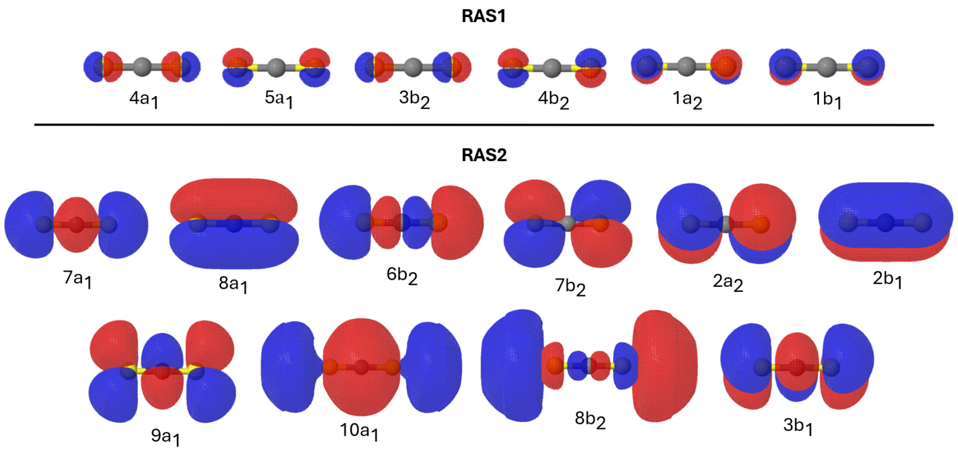

Figure S8: CS_2. Active space orbitals of the electronic ground state. The orbitals are SCF orbitals.

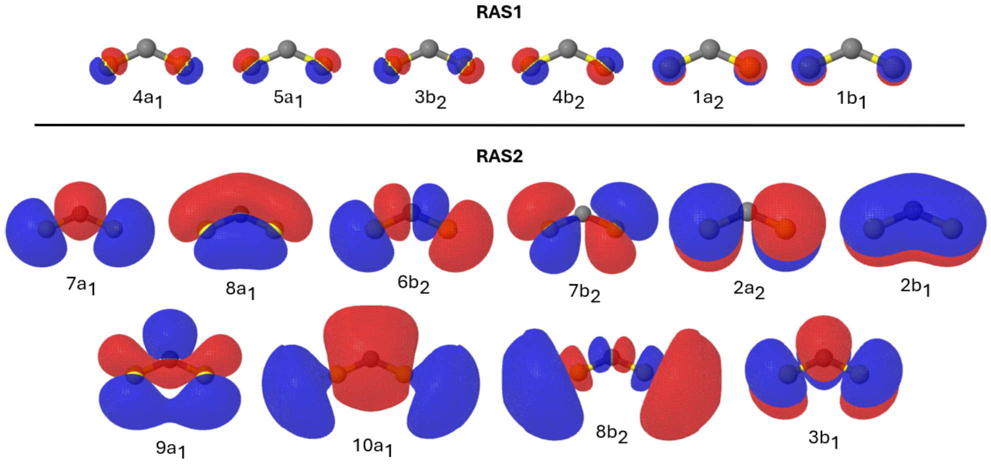

Figure S9: CS_2. Active space of 1 $^1B_2$ (at the optimized geometry of the state). The orbitals are SCF orbitals.

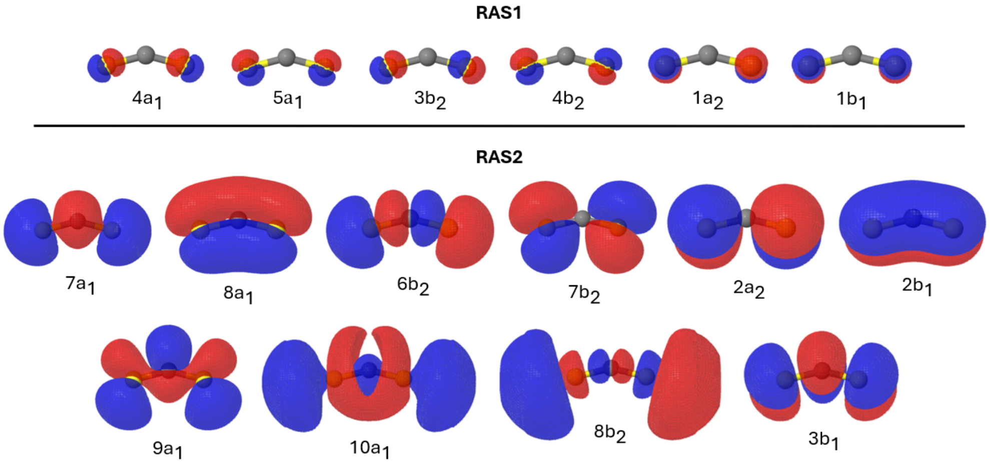

Figure S10: CS_2. Active space of 2 $^1B_2$ (at the optimized geometry of the state). The orbitals are SCF orbitals.

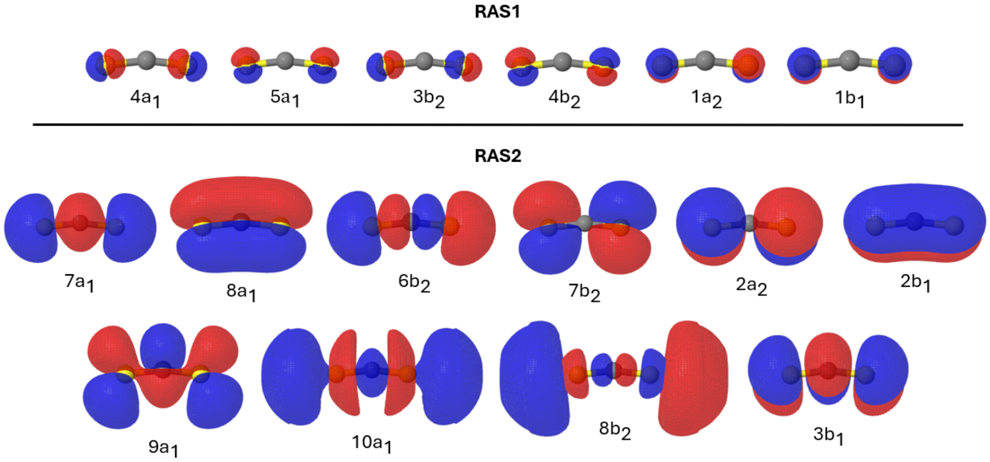

Figure S11: CS_2. Active space of 2 $^3A_2$ (at the optimized geometry of the state). The orbitals are SCF orbitals.

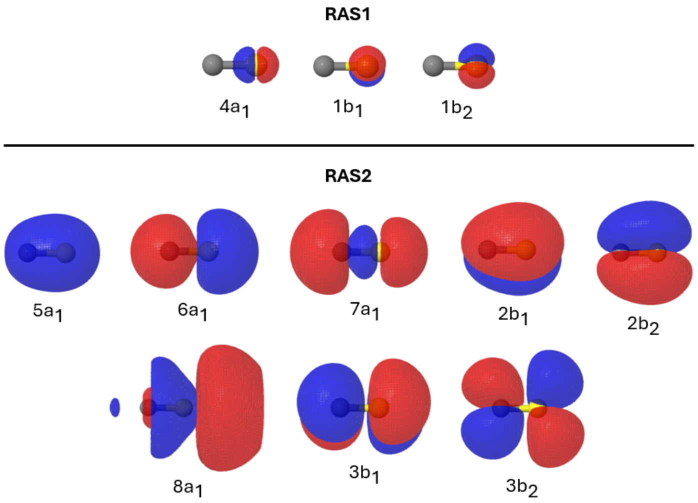

Figure S12: CS. Active space of the electronic ground state. The orbitals are SCF orbitals.

S3.2.2 RASSCF Orbitals

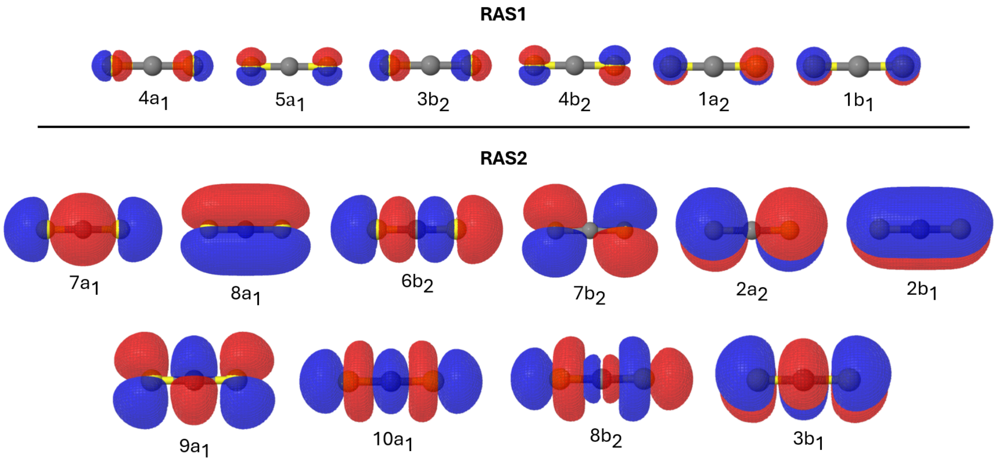

Figure S13: CS_2. Active space of the electronic ground state. RASSCF orbitals with SCF labels.

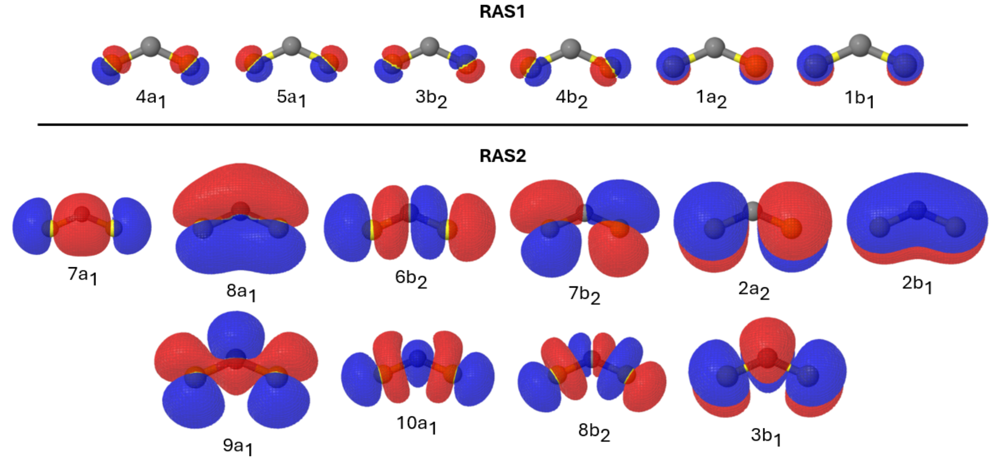

Figure S14: CS_2. Active space of 1 $^1B_2$ (at the optimized geometry of the state). RASSCF orbitals with SCF labels.

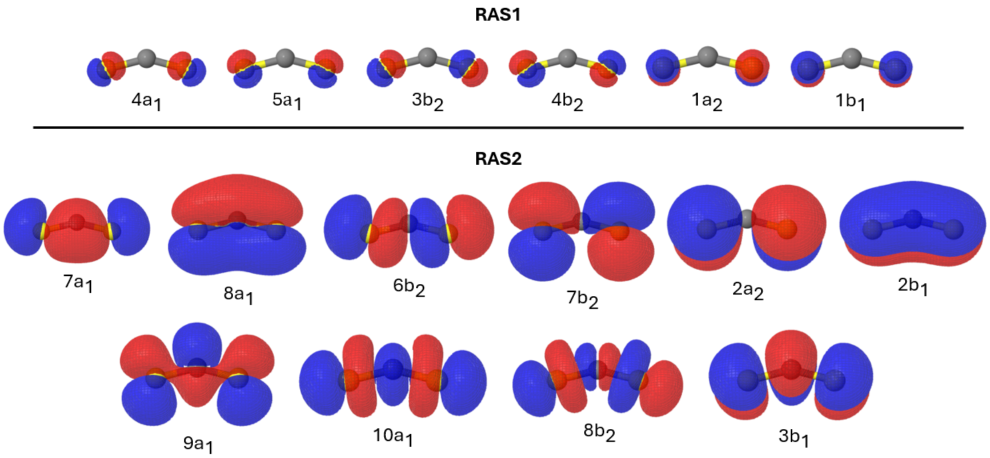

Figure S15: CS_2. Active space of 2 $^1B_2$ (at the optimized geometry of the state). RASSCF orbitals with SCF labels.

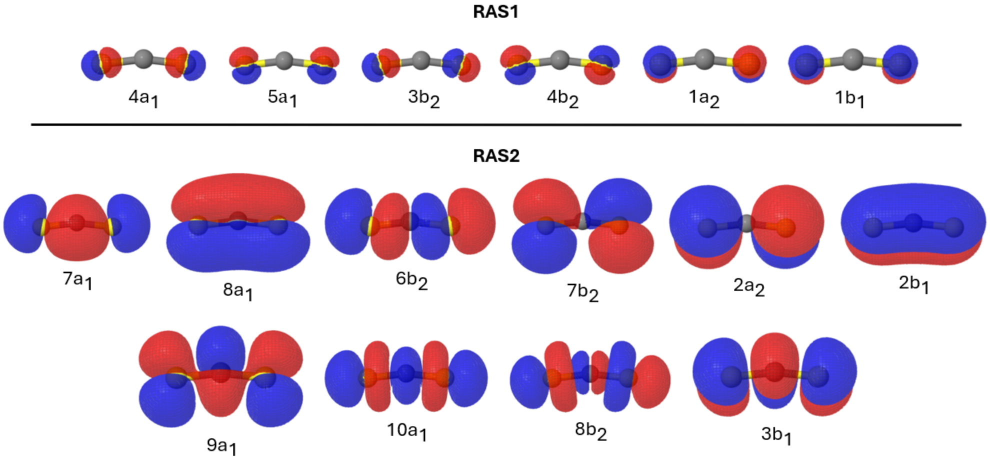

Figure S16: CS_2. Active space of 2 $^3A_2$ (at the optimized geometry of the state). RASSCF orbitals with SCF labels.

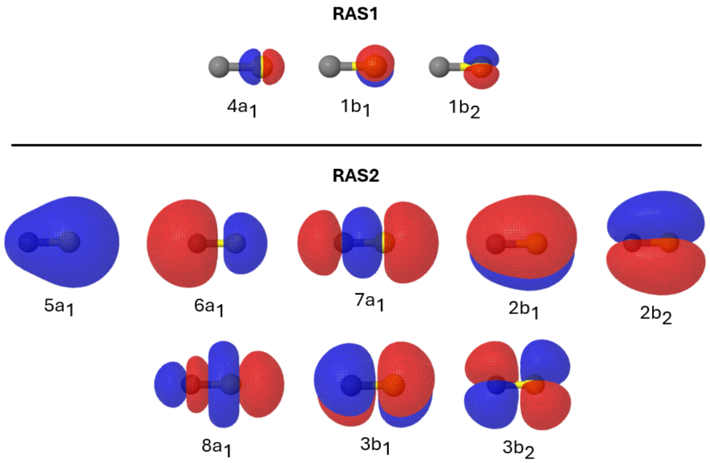

Figure S17: CS. Active space of the electronic ground state. RASSCF orbitals with SCF labels.

S3.3 Computed XPS spectra

We provide all the theoretical XPS spectra in Fig. S18. Each spectrum is computed using the optimized structure of the corresponding initial state. The transient XPS spectra of the valence-excited 2 $^1B_2$ $[^1\Sigma_u^+]$ state computed at both the Franck-Condon (FC) and the optimized (relaxed) excited-state structure are compared in Fig. S19.

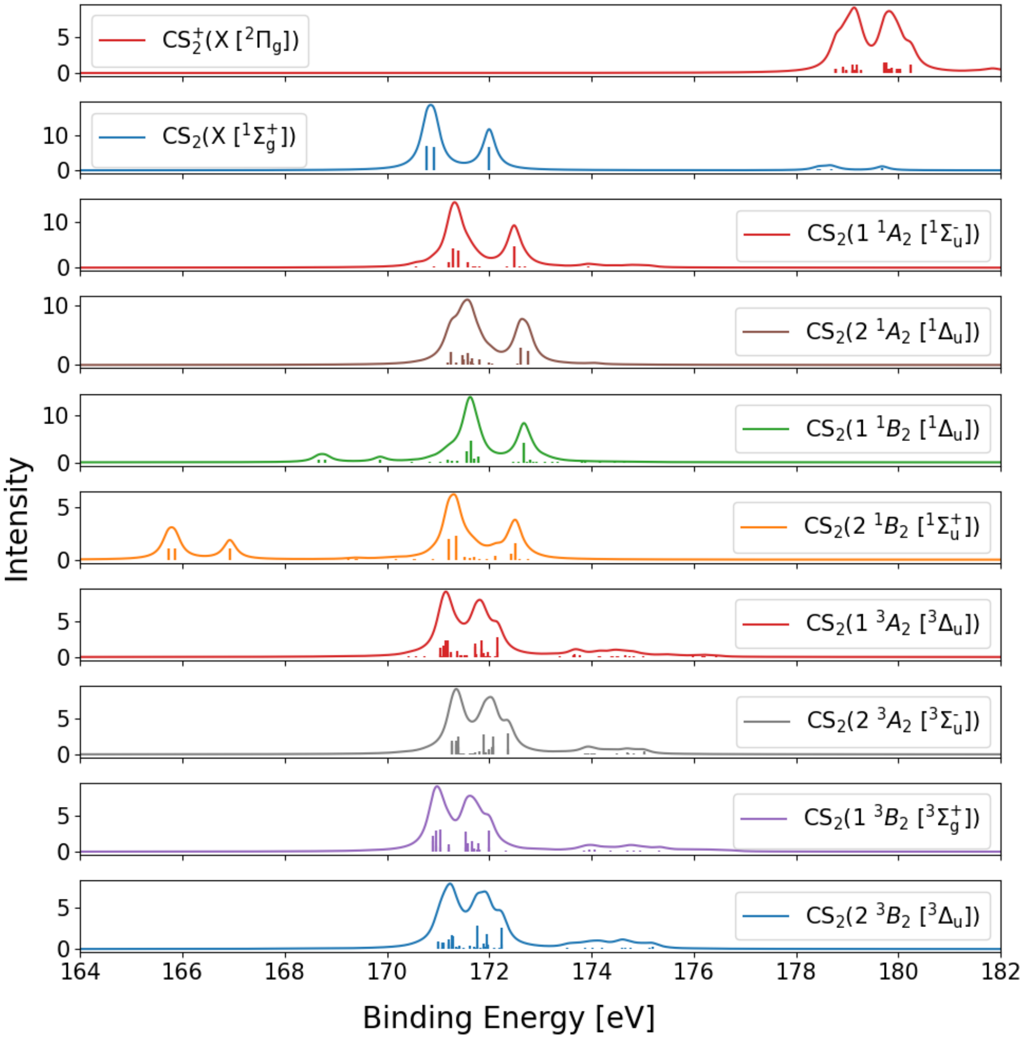

Figure S18: Computed XPS spectra of CS_2 and CS_2^+. The sticks have been broadened by Lorentzian functions with FWHM = 0.3 eV.

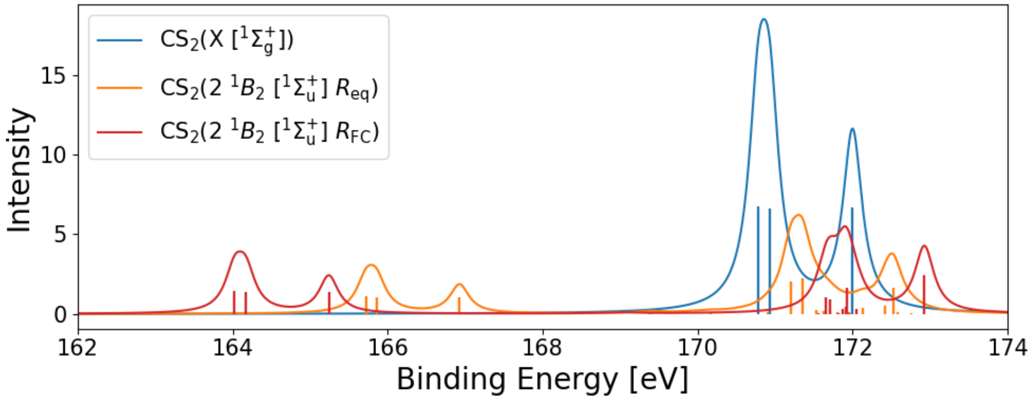

Figure S19: CS_2. Transient XPS spectrum of valence-excited (2 $^1B_2$ [$^1\Sigma_u^+$]) at both the Franck-Condon (FC) and the optimized (relaxed) excited-state structures. The sticks have been broadened by Lorentzian functions with FWHM = 0.3 eV.

S3.4 Assignment of spectroscopic features

In Tab. S8, we report the valence excitation energies and corresponding oscillator strengths computed at the MS-RASPT2 level of theory, together with the dominant configuration of the transition and the character assigned to the transition. The computed shake-down features are assigned in Tab. S9.

Table S8: CS_2. Computed MS-RASPT2 valence transitions from the ground state.

Final State	ΔE (eV)	f_{osc}	Configuration	Character
2 $^3A_2$ [$^3\Sigma_u^-$]	4.01	-	$(2a_2)^{-1}(9a_1)^1$ [0.44] + $(7b_2)^{-1}(3b_1)^1$ [0.44]	$\pi^* \leftarrow n$
1 $^1B_2$ [$^1\Delta_u$]	4.10	-	$(7b_2)^{-1}(9a_1)^1$ [0.43] + $(2a_2)^{-1}(3b_1)^1$ [0.43]	$\pi^* \leftarrow n$
2 $^1B_2$ [$^1\Sigma_u^+$]	6.79	1.25	$(7b_2)^{-1}(9a_1)^1$ [0.42] + $(2a_2)^{-1}(3b_1)^1$ [0.42]	$\pi^* \leftarrow n$

Table S9: CS_2. Computed MS-RASPT2 ground-state primary and valence excited-state shake-down features (binding energies (BE), averaged Dyson intensities (R_{FI}), and corresponding assignment). No shake-down features were found starting from the triplet valence-excited states with a threshold of 10^{-5} for the Dyson intensity.

BE (eV)	R_{FI}	Assignment
initial state: $\tilde{X}$ $^1A_1$ [$^1\Sigma_g^+$] R_{FC}		
170.784	0.664	$2p^{-1}$
170.928	0.660	$2p^{-1}$
171.999	0.662	$2p^{-1}$
initial state: 2 $^1B_2$ [$^1\Sigma_u^+$] R_{FC}		
164.027	0.142	$2p^{-1}$
164.170	0.138	$2p^{-1}$
165.242	0.138	$2p^{-1}$
initial state: 2 $^1B_2$ [$^1\Sigma_u^+$] R_{eq}		
165.723	0.108	$2p^{-1}$
165.856	0.106	$2p^{-1}$
166.928	0.106	$2p^{-1}$
initial state: 1 $^1B_2$ [$^1\Delta_u$] R_{eq}		
168.665	0.060	$2p^{-1}$
168.800	0.059	$2p^{-1}$
169.866	0.058	$2p^{-1}$

S3.5 Additional CCSD results

For comparison, we also performed coupled cluster singles and doubles (CCSD) calculations using the aug-cc-pVDZ basis set. All CCSD calculations were carried out with Q-Chem.[S8]

Table S10 collects the CCSD results for the valence excitation energies and oscillator strengths at the linear ground state geometry. The table also summarizes how the state labels change when changing the conventions for the molecular orientation and point group symmetry.

Figure S20 shows the natural transition orbitals (NTO) that characterize the first four singlet valence excited states of CS_2 at the linear geometry of the ground state (FC geometry). The NTOs of each of the first four singlet valence excited states at the respective valence excited state geometry are shown in Fig. S21.

Finally, Table S11 collects the CCSD/aug-cc-pVDZ excitation energies (and oscillator strengths for the singlet excitations) of the first four singlet and first five triplet excited states at FC geometry and their respective optimized minimum geometries.

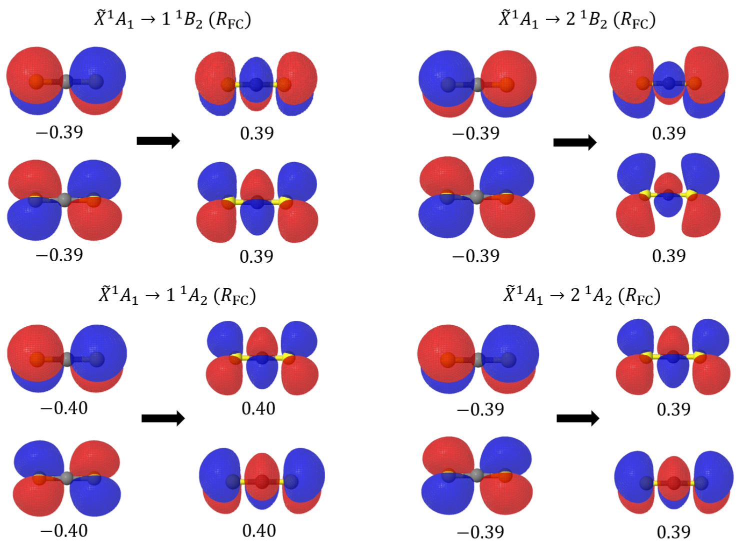

Figure S20: CCSD Natural transition orbitals of the first 4 valence excitations at the linear FC geometry (labelled R_{FC}). The numerical values are the weights of the shown orbital transition in the given NTO. Negative values are for hole orbitals and positive values for particle orbitals.

Table S10: CCSD/aug-cc-pVDZ excitation energies (ΔE) and oscillator strengths (f_{osc}) for the linear ground state geometry. Symmetry relabeling according to different orientations of the molecule in D_{2h} and according to C_{2v} (Mulliken's convention). In D_{2h}(z) [$D_{\infty h}$] the molecule is aligned along z. In D_{2h}(y) it is aligned along y. In C_{2v} it is aligned along y (such that the bent C_{2v} molecule lies on the yz plane with the z axis as the C_2 axis.

$D_{2h}(z)$ [$D_{\infty h}$]	ΔE (eV)	f_{osc} (dip)	D_{2h}(y)	C_{2v}(yz)
^1A_u [$^1\Sigma_\text{u}^-$]	4.12081	0.0000	^1A_u	$^1A_2$
$^1B_{1\text{u}}$ [$^1\Delta_\text{u}$]	4.15600	0.0000	$^1B_{2\text{u}}$	$^1B_2$
^1A_u [$^1\Delta_\text{u}$]	4.15600	0.0000	^1A_u	$^1A_2$
$^1B_{1\text{u}}$ [$^1\Sigma_\text{u}^+$]	6.53984	1.1739(z)	$^1B_{2\text{u}}$(y)	$^1B_2$(y)
$^1B_{2\text{g}}$ [$^1\Pi_\text{g}$]	6.92864	0.0000	$^1B_{1\text{g}}$	$^1B_2$
$^1B_{3\text{g}}$ [$^1\Pi_\text{g}$]	6.92864	0.0000	$^1B_{3\text{g}}$	$^1A_2$
$^1B_{1\text{g}}$ [$^1\Sigma_\text{g}^-$]	7.41577	0.0000	$^1B_{2\text{g}}$	$^1B_1$
$^1B_{1\text{g}}$ [$^1\Delta_\text{g}$]	7.51654	0.0000	$^1B_{2\text{g}}$	$^1B_1$
^1A_g [$^1\Delta_\text{g}$]	7.51654	0.0000	^1A_g	$^1A_1$
$^1B_{2\text{g}}$ [$^1\Pi_\text{g}$]	7.57573	0.0000	$^1B_{1\text{g}}$	$^1B_2$
$^1B_{3\text{g}}$ [$^1\Pi_\text{g}$]	7.57573	0.0000	$^1B_{3\text{g}}$	$^1A_2$
$^1B_{3\text{u}}$ [$^1\Pi_\text{u}$]	7.90498	0.0664(x)	$^1B_{3\text{u}}$(x)	$^1A_1$(z)
$^1B_{2\text{u}}$ [$^1\Pi_\text{u}$]	7.90498	0.0664(y)	$^1B_{1\text{u}}$(z)	$^1B_1$(x)

Table S11: CCSD/aug-cc-pVDZ results for the first 4 singlet and the first 5 triplet excited states at different geometries. C_{2v}(yz) symmetry. Symmetry labels follow Mulliken's convention.

	@FC		@min(1 $^1A_2$)		@min(1 $^1B_2$)		@min(2 $^1B_2$)		@min(2 $^1A_2$)	
1 $^1A_2$	4.12081	0.0000	2.20950	0.0000	1.82458	0.0000	2.72980	0.0000	3.69193	0.0000
1 $^1B_2$	4.15600	0.0000	2.38444	0.0258	1.92929	0.0274	2.92306	0.0133	3.71273	0.0000
2 $^1A_2$	4.15600	0.0000	3.14745	0.0000	2.92882	0.0000	3.31698	0.0000	3.71488	0.0000
2 $^1B_2$(y)	6.53984	1.1739	4.80220	0.4509	4.57333	0.3648	5.04621	0.5782	6.04899	1.1253
	@FC		@min(1 $^3A_2$)		@min(1 $^3B_1$)		@min(2 $^3B_2$)		@min(2 $^3A_2$)	
$^3B_2$	3.42464		1.43616		0.66059		3.00244		2.93212	
$^3A_2$	3.81557		1.92986		1.35161		3.35497		3.27941	
$^3B_2$	3.81557		2.98703		**2.8521**		3.38450		3.33690	
$^3A_2$	4.12345		3.00346		2.66332		3.65409		3.60005	
$^3A_1$	6.22134		4.20605		3.85602		5.35411		5.27012	

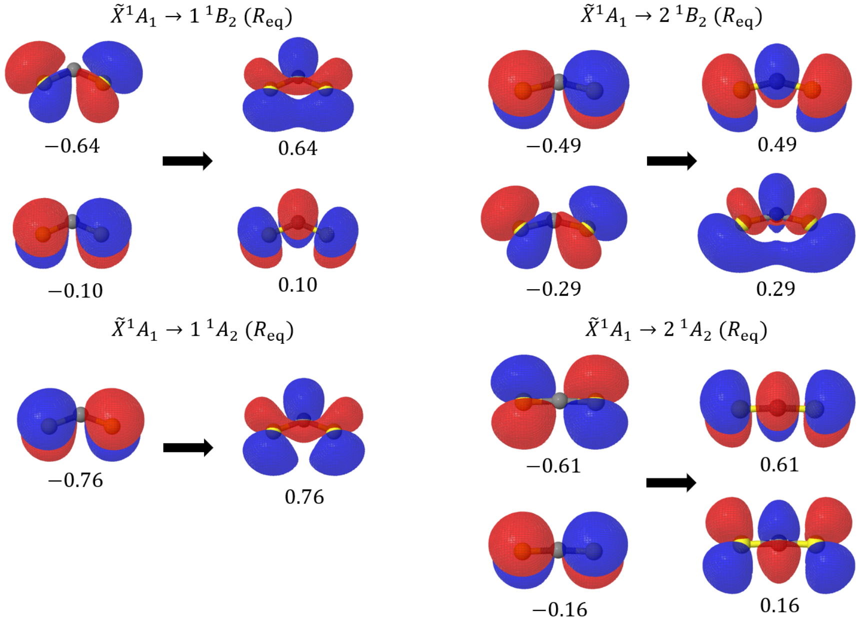

Figure S21: CCSD Natural transition orbitals of the first 4 valence excitations at their respective optimized geometry (labelled R_{eq}). The numerical values are the weights of the shown orbital transition in the given NTO. Negative values are for hole orbitals and positive values for particle orbitals.

References

(S1) Hedin, L.; Eland, J. H. D.; Karlsson, L.; Feifel, R. An x-ray absorption and a normal Auger study of the fine structure in the $S2p^{-1}$ region of the CS_2 molecule. J. Phys. B: At. Mol. Opt. Phys. **2009**, 42, 085102.

(S2) Wang, H.; Bässler, M.; Hjelte, I.; Burmeister, F.; Karlsson, L. A vibrationally resolved experimental study of the sulfur L-shell photoelectron spectrum of the CS_2 molecule. J. Phys. B: At. Mol. Opt. Phys. **2001**, 34, 1745.

(S3) Baltzer, P.; Wannberg, B.; Lundqvist, M.; Karlsson, L.; Holland, D.; MacDonald, M.; Hayes, M.; Tomasello, P.; von Niessen, W. An experimental and theoretical study of the valence shell photoelectron spectrum of carbon disulphide. Chem. Phys. **1996**, 202, 185–209.

(S4) Kandula, D. Z.; Gohle, C.; Pinkert, T. J.; Ubachs, W.; Eikema, K. S. E. Extreme Ultraviolet Frequency Comb Metrology. Phys. Rev. Lett. **2010**, 105, 063001.

(S5) Smith, A. D.; Warne, E. M.; Bellshaw, D.; Horke, D. A.; Tudorovskya, M.; Springate, E.; Jones, A. J. H.; Cacho, C.; Chapman, R. T.; Kirrander, A. et al. Mapping the Complete Reaction Path of a Complex Photochemical Reaction. Phys. Rev. Lett. **2018**, 120, 183003.

(S6) Karashima, S.; Suzuki, Y.-I.; Suzuki, T. Ultrafast Extreme Ultraviolet Photoelectron Spectroscopy of Nonadiabatic Photodissociation of CS_2 from $^1B_2(^1\Sigma_u^+)$ State: Product Formation via an Intermediate Electronic State. J. Phys. Chem. Lett. **2021**, 12, 3755–3761.

(S7) Brown, S. T.; Van Huis, T. J.; Hoffman, B. C.; Schaefer, H. F., III Excited electronic states of carbon disulphide. Mol. Phys. **1999**, 96, 693–704.

(S8) Epifanovsky, E.; Gilbert, A. T. B.; Feng, X.; Lee, J.; Mao, Y.; Mardirossian, N.; Pokhilko, P.; White, A. F.; Coons, M. P.; Dempwolff, A. L. et al. Software for the frontiers of quantum chemistry: An overview of developments in the Q-Chem 5 package. J. Chem. Phys. **2021**, 155, 084801.

(S9) Huber, K.-P.; Herzberg, G. Molecular Structure Constants of Diatomic Molecules; Van Nostrand Reinhold: New York, 1979.

(S10) Jmol: an open-source Java viewer for chemical structures in 3D. `http://www.jmol.org`.
